# NucleoMap: A computational tool for identifying nucleosomes in ultra-high resolution contact maps

**DOI:** 10.1371/journal.pcbi.1010265

**Published:** 2022-07-14

**Authors:** Yuanhao Huang, Bingjiang Wang, Jie Liu

**Affiliations:** 1 Department of Computational Medicine & Bioinformatics, University of Michigan, Ann Arbor, Michigan, United States of America; 2 Department of Computer Science & Engineering, University of Michigan, Ann Arbor, Michigan, United States of America; Heidelberg University, GERMANY

## Abstract

Although poorly positioned nucleosomes are ubiquitous in the eukaryotic genome, they are difficult to identify with existing nucleosome identification methods. Recently available enhanced high-throughput chromatin conformation capture techniques such as Micro-C, DNase Hi-C, and Hi-CO characterize nucleosome-level chromatin proximity, probing the positions of mono-nucleosomes and the spacing between nucleosome pairs at the same time, enabling nucleosome profiling in poorly positioned regions. Here we develop a novel computational approach, NucleoMap, to identify nucleosome positioning from ultra-high resolution chromatin contact maps. By integrating nucleosome read density, contact distances, and binding preferences, NucleoMap precisely locates nucleosomes in both prokaryotic and eukaryotic genomes and outperforms existing nucleosome identification methods in both precision and recall. We rigorously characterize genome-wide association in eukaryotes between the spatial organization of mono-nucleosomes and their corresponding histone modifications, protein binding activities, and higher-order chromatin functions. We also find evidence of two tetra-nucleosome folding structures in human embryonic stem cells and analyze their association with multiple structural and functional regions. Based on the identified nucleosomes, nucleosome contact maps are constructed, reflecting the inter-nucleosome distances and preserving the contact distance profiles in original contact maps.

## Author summary

Nucleosomes are the conservative building blocks of the chromatin, and their array regularity and positioning level correlate with transcription activity, but their underlying distributions in eukaryote genomes have not been comprehensively studied due to the poorly positioned ones. Recently available high-throughput enhanced chromatin conformation capture techniques such as Micro-C, DNase Hi-C, and Hi-CO provide information of nucleosome-level chromatin proximity, including the positions of mononucleosomes and the spacing between nucleosome pairs, enabling identifying nucleosomes in poorly positioned regions. Here, we present NucleoMap, a nucleosome identification approach from ultra-high resolution chromatin contact maps. In this paper, we provide an overview of NucleoMap’s workflow and capabilities. We benchmark NucleoMap with popular baseline methods in multiple datasets. Next, we rigorously characterize genome-wide association between the spatial organization of mono-nucleosomes and their corresponding histone modifications, protein binding activities, and higher-order chromatin functions in eukaryotes. Based on the identified nucleosomes, we also construct more precise and more interpretable nucleosome contact maps, which preserve the inter-nucleosome distances.

## Introduction

Nucleosomes are the conservative building blocks of the hierarchical chromatin structure, on which higher-order structures are formed [1]. Genome-wide approaches revealed that nucleosomes are regularly spaced and organized into arrays on a single chromatin fiber, but the variation between fibers, defined as the positioning level, may vary in different species [2]. Almost all nucleosomes are well-positioned in yeast, meaning that the arrays occupy the same location of the chromatin fiber in the majority of a cell population [3], but the positions of the nucleosome arrays are much more flexible (poorly-positioned) in animals and plants [4–6]. The positioning level of nucleosomes are reflected by the patterns of chromatin accessibility, which is captured by sequencing techniques such as MNase-seq [7], DNase-seq [8], and ATAC-seq [9]. By definition, well-positioned nucleosomes are stable between chromatin fibers and thus yield narrow peaks. On the contrary, poorly-positioned nucleosomes have broad and flat peaks.

Current nucleosome identification methods rely on calling peaks from MNase-seq [10–13], ChIP-seq [14, 15], or ATAC-seq data [16, 17]. However, because the patterns of neighboring broad peaks are largely overlapped, it is difficult to locate poorly-positioned nucleosomes accurately using these methods [4, 18]. One promising approach is to separate the merged signal into single peaks using the nucleosome repeat length (NRL) [19]. NRL is the average distance between the centers of neighboring nucleosomes, which remains unchanged within a nucleosome array. By matching the distribution of local NRLs, it is possible to align the nucleosome arrays in poorly-positioned regions. Therefore, in addition to the mono-nucleosome occupancy along the chromatin fiber captured by the aforementioned sequencing techniques, it is necessary to integrate information regarding inter-nucleosome distances from other data sources.

Recently available high-throughput enhanced chromatin conformation capture (Hi-C) techniques such as Micro-C [20–22], DNase Hi-C [23] and Hi-CO [24] provide information of nucleosome-level chromatin proximity. These ultra-high resolution chromatin contact map data capture both *mono-nucleosomes’ positions* characterized by the read alignments and the *spacing between nucleosome pairs* characterized by contact distances. Integrating nucleosome positioning and spacing information enables identifying nucleosomes in poorly-positioned regions. With the increasing availability of the data, identifying nucleosomes from ultra-high resolution chromatin contact maps becomes meaningful. However, no computational approach has been specifically designed to identify nucleosome positions from ultra-high resolution chromatin contact maps to our best knowledge.

In this work, we present NucleoMap, a nucleosome position characterization approach from ultra-high resolution chromatin contact maps. By integrating genomic sequence specificity, read density, and pairing information, NucleoMap precisely locates both well-positioned nucleosomes and poorly-positioned nucleosomes, outperforming existing nucleosome identification methods in both precision and recall. We rigorously characterize genome-wide association in eukaryotes between the spatial organization of mono-nucleosomes and their corresponding histone modifications, protein binding activities, and higher-order chromatin functions. We find evidence of two tetra-nucleosome folding motifs, *α*-tetrahedron and *β*-rhombus, in human embryonic stem cells. The association between preferences on folding motifs and genome structure is investigated. Based on the identified nucleosomes, nucleosome contact maps are constructed, which preserve the inter-nucleosome distances. In this way, nucleosome contact maps capture the original contact distance profile, making them more concentrated and more interpretable than traditional fixed-bin-based contact maps.

## Results

### NucleoMap algorithm

Existing methods detect nucleosomes either by identifying genomic regions with enriched reads [11–14] or by calculating normalized nucleosome occupancy profiles [10, 15]. As a result, these models are not sensitive to identifying nucleosomes in poorly-positioned regions where peaks are broad and largely overlapped. To overcome the limitation, we develop an approach called NucleoMap, separating neighboring peaks using local NRLs. NucleoMap identifies nucleosomes at different positioning levels from ultra-high resolution chromatin contact maps, including Micro-C [20, 21], DNase Hi-C [23] and Hi-CO [24]. Different from MNase-seq or ATAC-seq, these ultra-high resolution chromatin contact maps capture both the positions of mono-nucleosomes and the inter-nucleosome distances on the chromatin fiber, allowing modeling nucleosome occupancy and local NRLs at the same time.

**Fig 1:**
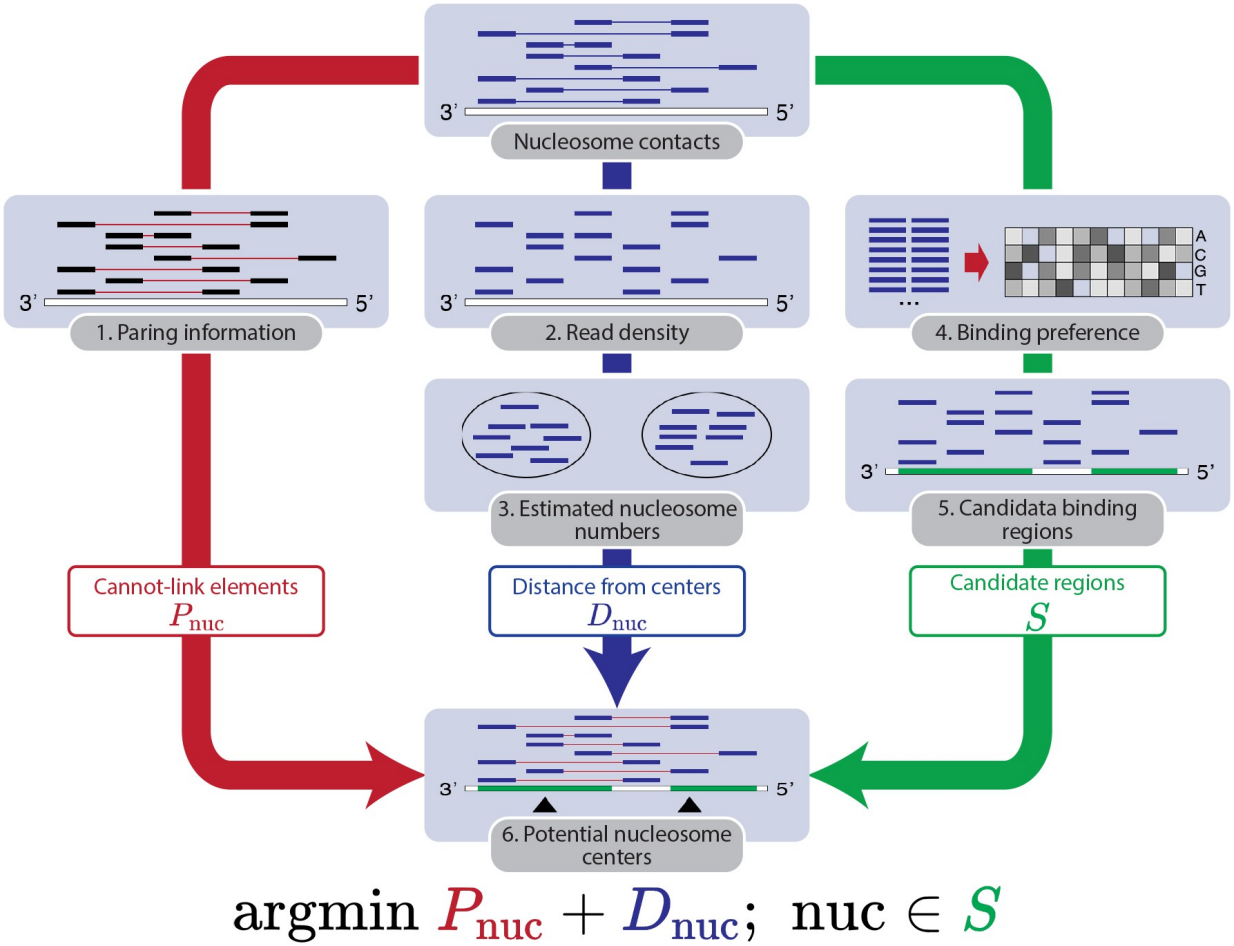
Workflow of the NucleoMap model. NucleoMap locates nucleosome centers with the following steps: 1. Extract the pairing information of reads from ultra-high resolution chromatin contact maps. Two ends from the same contact are assigned to different nucleosomes. 2. Extract aligned reads from ultra-high resolution chromatin contact maps. 3. Estimate the number of nucleosomes from the aligned reads using a Dirichlet prior. 4. Calculate nucleosome-binding preferences from the contact sequences. 5. Identify candidate nucleosome-binding regions with the binding preference in the previous step. 6. Calculate nucleosome centers in candidate binding regions by integrating the read positions and pairing information.

NucleoMap uses a parametric model to separate the read density into multiple local distributions, each representing the positioning of an individual nucleosome. In particular, every nucleosome’s position is explicitly characterized by a Gaussian distribution, and NucleoMap identifies them by minimizing an objective function integrating the distribution of read alignments, inter-nucleosome distances, and nucleosome binding preferences (Fig 1).

The first information is the aligned read density, which is also used by traditional peak-calling approaches (Fig 1 step 2). Based on the fact that every alignment (one end of a contact) represents a mono-nucleosome from an individual cell, NucleoMap optimizes the expected positions of mono-nucleosomes following a constrained *k*-means paradigm. Due to the unknown number of nucleosomes in a region, the value of *k* is adaptively determined using a Dirichlet process (DP) prior. As a result, the positions of nucleosomes are defined as the mean of local read densities (Fig 1 step 3).

Uniquely captured by ultra-high resolution contact maps, the inter-nucleosome distances are also utilized to adjust the positions of identified nucleosomes (Fig 1 step 1). To ensure that the distance between neighboring nucleosomes resembles local NRLs, two ends of every contact are assigned to different nucleosomes. Specifically, two ends of every contact are considered as cannot-link elements in the constrained *k*-means optimization (Fig 1 step 6) [25]. In this way, the distances between identified nucleosome centers are adjusted by the inter-nucleosome distances from the data.

The third piece of relevant information comes from nucleosome binding preference reflected by the nucleosome binding motifs (Fig 1 step 4). It is known that nucleosomes are enriched for particular DNA sequence motifs on the nucleosomal DNA, most notably ∼10bp periodic occurrences of AA/AT/TA/TT 2-mers [26–28]. NucleoMap models the AA/AT/TA/TT dinucleotide motifs using a dinucleotide position weight matrix (PWM) calculated from the aligned reads (S1 Fig), and then calculates a motif-based nucleosome-binding score along the genome using the dinucleotide PWM. By integrating the binding score as a penalty term in the computation of distances, NucleoMap considers the sequence specificity in nucleosome identification (Fig 1 step 6).

Using an objective function integrating the three aforementioned types of information, NucleoMap characterizes mono-nucleosome positions by solving a constrained *k*-means problem with a Dirichlet prior. In the end, the reads are separated into different clusters representing mono-nucleosomes, while the number of nucleosomes *k* is automatically learned using a hyperparameter λ. λ controls the fuzziness threshold of nucleosome calling.

### NucleoMap accurately locates well-positioned and poorly-positioned nucleosomes

To the best of our knowledge, no computational approach has been specifically designed to identify nucleosome positions from ultra-high resolution chromatin contact maps. To evaluate the performance achieved by our method, we compare NucleoMap with four popular nucleosome callers designed for MNase-seq data [10, 11, 13, 29], and the Micro-C contact maps are treated as single-end MNase-seq data by ignoring the pairing information between alignments.

**Fig 2:**
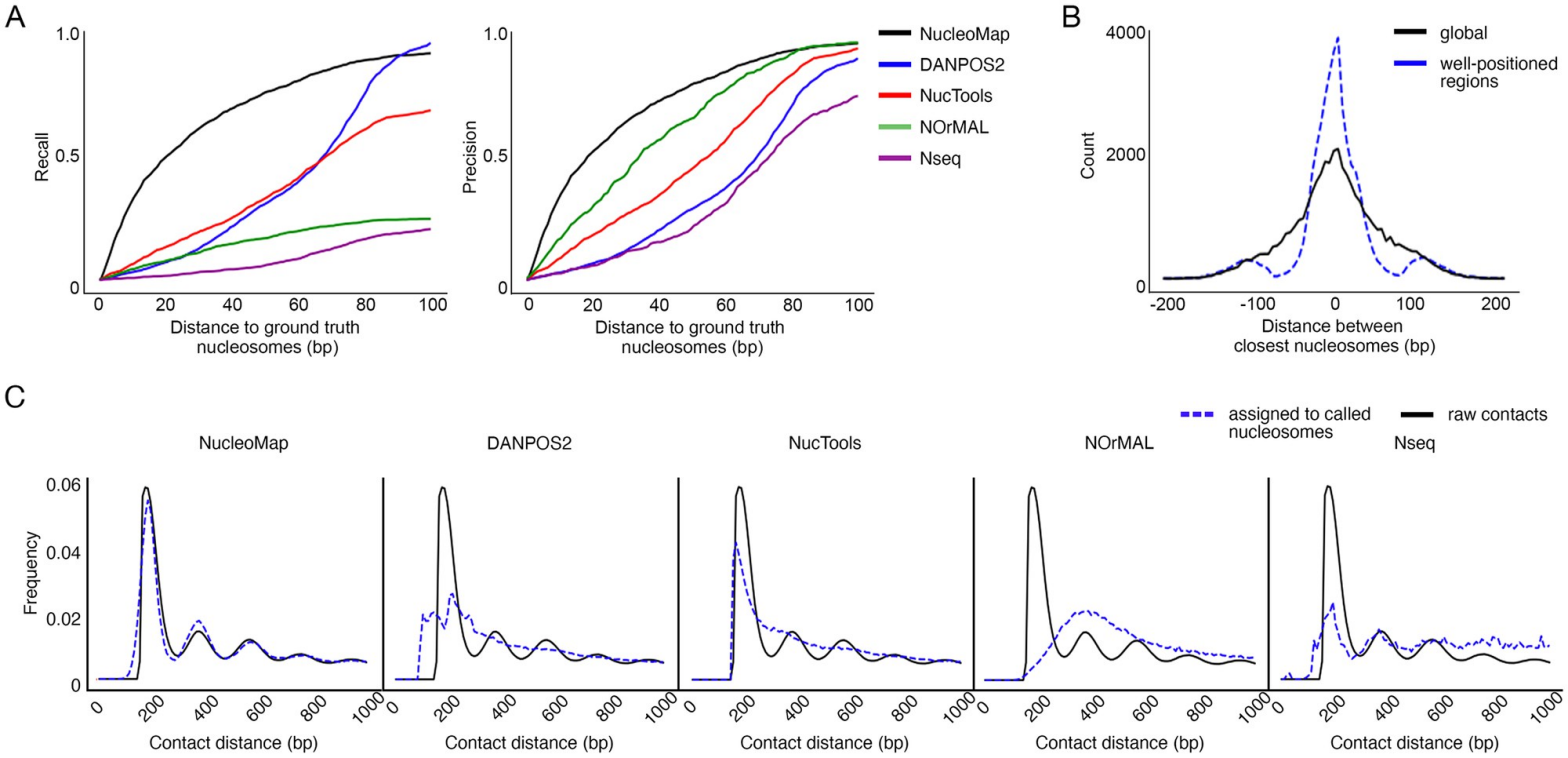
NucleoMap outperforms baseline methods in yeast and hESC. NucleoMap locates nucleosome centers with the following steps: A. (left panel) Recall of nucleosomes identified by different approaches against the corresponding distance thresholds in yeast chrIII. The recall is calculated by n(true-positive nucleosomes)/n(ground truth nucleosomes). Here “true-positive” nucleosomes refer to nucleosomes located within certain distance thresholds from a “ground truth” nucleosome, while the “ground truth” nucleosomes are experimentally confirmed nucleosomes. Smaller distance thresholds correspond to more accurate nucleosome locations, while a higher recall corresponds to more identified ground truth nucleosomes. Therefore, the area under the curve represents the sensitivity of the corresponding methods in identifying nucleosomes (right panel). The precision of nucleosomes identified by different approaches against the corresponding distance threshold in yeast chrIII. Precision is calculated by n(true-positive nucleosomes)/n(identified nucleosomes). A higher consensus nucleosome ratio represents fewer “false-positive” nucleosomes identified, and thus the area under the curves represents the nucleosome identifying specificity of the corresponding methods. B. Distance between nucleosomes identified by NucleoMap (NucleoMap nucleosomes) and their nearest nucleosomes identified by DANPOS2 (DANPOS2 nucleosomes) in different regions. Compared with random regions in the whole genome, NucleoMap nucleosomes are much closer to the nearest DANPOS2 nucleosomes in well-positioned regions, showing the consistency in well-positioned regions across the two methods. C. Histogram of contact distance (black) and histogram of inter-nucleosome distance characterized by computational methods (blue) in hESC chr21. The peak patterns of contact distance reflect genome-wide nucleosome repeating lengths (NRL). Similar histograms between raw contact distance and computationally characterized inter-nucleosome distance suggest accurate nucleosome identification.

We first compare the precision and recall of nucleosome calling in yeast, where the positions of nucleosomes are experimentally confirmed [28]. Using these experimentally confirmed nucleosomes as the ground truth, the evaluation criteria are calculated as follows. First, the distance between a nucleosome position identified by the caller and its nearest experimentally confirmed nucleosome *d* is calculated. Then, nucleosomes with *d* ≤ *d*_*t*_ are considered to be true-positive, where *d*_*t*_ is a certain threshold. True-positives represent nucleosomes that are validated by the experiment. In the end, the precision and recall are calculated for every method under different distance thresholds. We have the following observations. First, NucleoMap achieves the highest recall at *d*_*t*_ ≤90bp, and it has the second-highest recall when distance threshold *d*_*t*_ > 90bp (Fig 2A). Compared with baseline methods, NucleoMap identifies a larger number of ground truth nucleosomes with *d*_*t*_ ≤90bp, measured by the areas under the curves, suggesting its higher sensitivity in accurately identifying nucleosomes. At *d*_*t*_ = 100bp, almost all ground truth nucleosomes are recovered by NucleoMap and DANPOS2, which recognize 1,554 and 1,622 out of 1,716 ground truth nucleosomes respectively. In comparison, 1,492 ground truth nucleosomes are identified by nucleR, 417 identified by NOrMAL and 346 identified by Nseq. Second, NucleoMap has the highest precision when distance threshold *d*_*t*_ < 80bp and the second-highest precision when *d*_*t*_ > 80bp. Compared with baseline methods, NucleoMap identifies the second-largest proportion of ground truth nucleosomes, suggesting a low false-positive rate achieved by our method. Almost all nucleosomes identified by NucleoMap (95.2%) are ground truth nucleosomes at *d*_*t*_ = 100bp. Ground truth nucleosomes identified by nucleR (98.1%) and NOrMAL (95.1%) also account for a large proportion, followed by DANPOS2 (88.9%) and Nseq (73.8%). Therefore, NucleoMap achieves comparable or better performance than baseline models in both precision and recall.

To further demonstrate that our method identifies both well-positioned and poorly-positioned nucleosomes, we compare the nucleosomes identified by NucleoMap and DANPOS2 in well-positioned regions that are known to us. In total, 42,679 identified nucleosomes in well-positioned regions are considered. As a control, we randomly select the same number of nucleosomes from the whole genome. In three types of well-positioned regions (promoters, insulators, and enhancers), nucleosomes identified by NucleoMap are significantly closer (∼ 50%) to their closest neighbors identified by DANPOS2, compared with the random control. The average distance between nucleosomes identified by NucleoMap and their closest neighbors identified by DANPOS2 in well-positioned regions is ∼ 20bp (Fig 2B). This result suggests that NucleoMap performs at least as good as, if not better than, existing nucleosome calling methods in well-positioned regions.

Finally, to examine the overall performance of our method in the more complex eukaryotic genomes, we compare the recovered contact profile from the callers with the original contact profile in hESC Micro-C data. The original contact profile is a histogram of contact distance, while the recovered contact profile is the histogram of inter-nucleosome distances between the assigned nucleosome centers. This profile reflects the real nucleosome spacing in the genome. In eukaryotic genomes where most nucleosomes are poorly-positioned, this comparison effectively evaluates the accuracy of poorly-positioned nucleosome arrays identified by computational methods. We calculate their recovered contact profiles in two steps. First, two ends of a read are assigned to their nearest called nucleosomes, forming a recovered contact, and the distance between the assigned nucleosome pair is considered as the recovered contact distance. Next, the recovered contact profile is built using these recovered contacts, illustrating the spacing between computationally identified nucleosomes. Finally, the recovered contact profile is compared to the original contact profile. If the nucleosome spacing is consistent between the nucleosomes identified by callers and the underlying ground truth nucleosomes in the data, the two profiles are similar to each other. Compared with the nucleosomes called by baseline methods, the recovered contact profile produced by NucleoMap is more similar to the original contact profile (Fig 2C). This result implies that NucleoMap achieves high accuracy in identifying nucleosomes in eukaryotic genomes.

### Nucleosome positioning level and spatial organization reflect patterns of histone modification and genome functions

It has been discovered that nucleosome positioning reflects the genome functions in different regions because the nucleosomes are directly decorated, composed, or impeded by specific histone variants and regulatory proteins [30, 31]. To further validate the identified nucleosomes, as well as to evaluate the connection between nucleosome spatial distribution and genome functions, nucleosome positioning levels and local nucleosome organization are compared at different epigenetic marks and transcriptional factor binding sites and in different genome functions.

**Fig 3:**
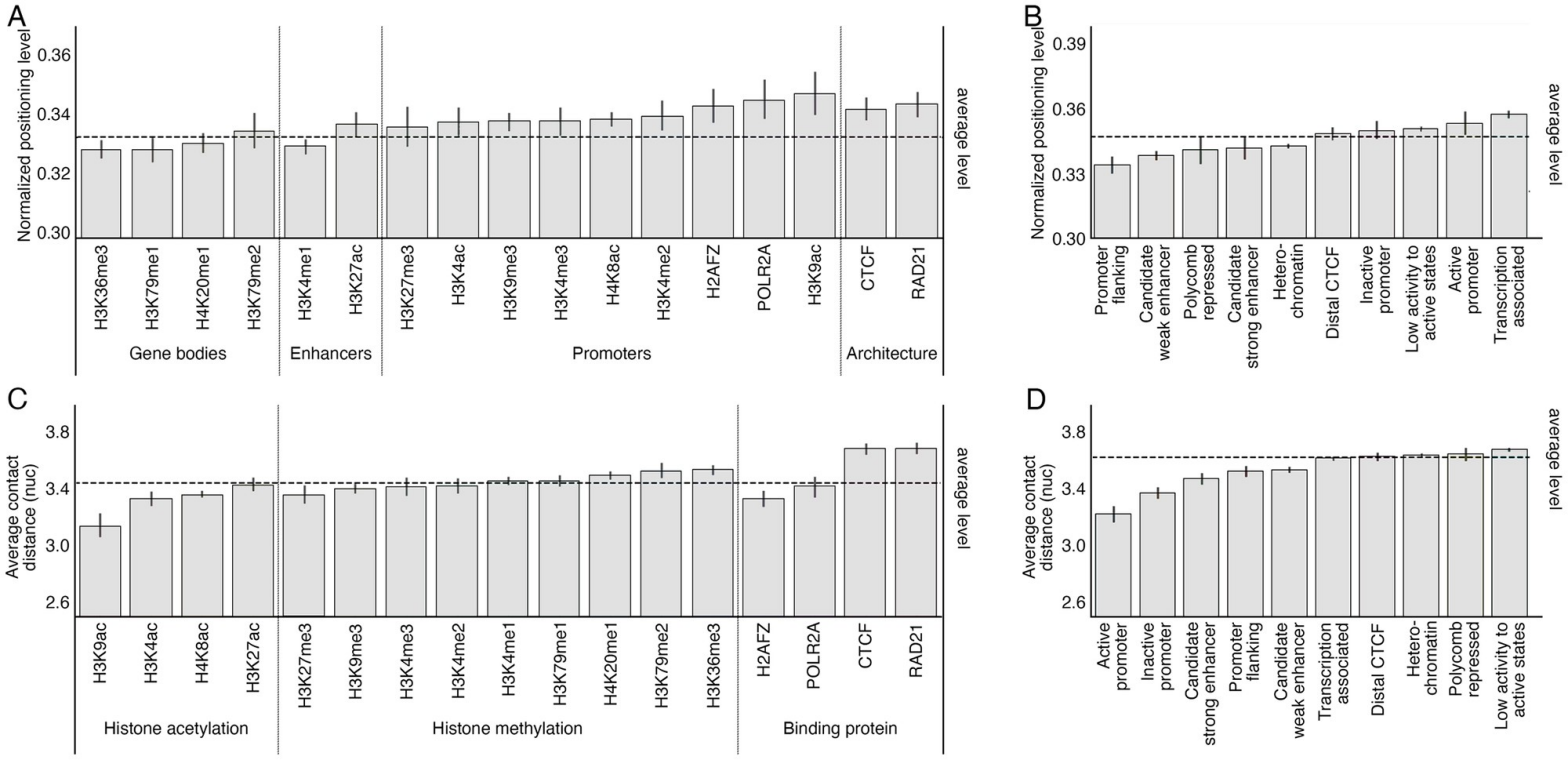
Nucleosome positioning levels and spatial organization are correlated with patterns of epigenetic modifications and genome functions. A. Normalized nucleosome positioning level at nucleosomes subject to specific epigenetic modifications or protein bindings. Generally, nucleosomes modified by promoter enriched epigenetic marks or at chromatin architecture associated protein binding sites are better positioned. Meanwhile, nucleosomes modified by gene body enriched or enhancer enriched epigenetic marks are more poorly positioned. B. Normalized nucleosome positioning level at nucleosomes within different chromatin states predicted by ChromHMM. Similar to the observation in epigenetic modifications, nucleosomes in promoters are better positioned, while nucleosomes in promoter flanking regions and enhancers are more poorly positioned. Transcription associated state represents loci of RNA polymerase binding or mRNA elongation, which mostly occur near active promoters. C. Average distances of local contacts (contact distances ≤ 1kb) at nucleosomes subject to specific epigenetic modifications or protein bindings. A longer contact distance suggests more compact nucleosome spatial organization, while a shorter contact distance suggests more relaxed nucleosome spatial organization. Generally, nucleosomes modified by histone methylations are more tightly packed than nucleosomes modified by histone acetylations, and the spatial organization of nucleosomes at protein binding sites varies across different protein functions. D. Average distances of local contacts (contact distances ≤ 1kb) at nucleosomes within different chromatin states predicted by ChromHMM. Compared with other states, nucleosomes at enhancers and promoters are more lightly packed regardless of their activities, resulting in shorter average contact distances.

Consistent with the existing conclusions [32, 33], we observe that nucleosome positioning levels at epigenetic marks and transcriptional factor binding sites better correlate with location rather than the regulatory direction (up-regulate or down-regulate) of the epigenetic binding (Fig 3A). We use a previously proposed measure called nucleosome occupancy to quantify the normalized nucleosome positioning level [18]. In general, modified nucleosomes at promoters tend to have higher positioning levels, followed by enhancers and gene bodies. Within a particular region, the positioning level at activation modification is slightly higher than repression modification, i.e., H3K9ac and H3K4me2 compared with H3K9me3 and H3K27me3 in promoter regions. Besides histone modifications, histone variants and all tested chromatin-binding proteins are also associated with higher positioning levels. For example, nucleosomes at structural proteins such as CTCF and RAD21 binding sites have higher positioning levels than the genome-wide average level. A consistent trend is confirmed by the nucleosome positioning levels in different chromatin states annotated by ChromHMM (Fig 3B). In promoters and transcription-active regions, nucleosomes are better positioned, whereas in enhancers and repressed regions such as polycomb repressed regions and heterochromatin, they are more poorly positioned.

To investigate the association between local nucleosome organization and genome functions, we calculate the average genomic distance of local contacts (i.e., contacts within 1kb) measured by nucleosomes at individual genomic regions, and compare the average genomic distances at different types of genomic regions. In general, a shorter average contact distance indicates a more relaxed chromatin fiber structure, while a longer average contact distance indicates a more compact chromatin fiber structure (S2 Fig). Compared with histone methylated regions, histone acetylated regions tend to correlate with a shorter average contact distance (Fig 3C). This result is consistent with the fact that histone acetylation is enriched at euchromatin where the chromatin fiber is lightly packed. Meanwhile, the influences of binding proteins on nucleosome spatial organization are various. Long average contact distances are observed at structural protein binding sites such as CTCF and RAD21, consistent with the previous studies that these proteins mediate chromatin looping and other structures [34]. On the contrary, short average contact distances are observed at transcription-associated protein POLR2A and histone variant H2AFZ. These factors are enriched at active TSSs, consistent with the fact that euchromatin with relaxed structures is enriched with expressed genes [35, 36]. In addition, nucleosome spatial organization is also correlated with chromatin states and transcription activities (Fig 3D). Short average contact distances are observed in promoters, enhancers, and the promoter flanking regions, indicating the chromatin is loose in these regions. Furthermore, average contact distances at active promoters are shorter than inactive ones, and strong enhancers are shorter than weak ones, suggesting that the nucleosomes are more lightly packed in regions more associated with transcription events.

### Tetra-nucleosome structural motifs closely correlate with genome functions and chromatin structures

Although global structural motifs have been confirmed in the contact profiles (or decaying curves) of ultra-high resolution contact maps in mouse [22], the location of structural motifs across the whole genome has not yet been studied because most nucleosomes are poorly-positioned. Recently, two types of tetra-nucleosome structural motifs, *α*-tetrahedron and *β*-rhombus, are discovered in yeast chromatin contact maps [24, 37]. Evidence of these folding motifs is also reported in human by electronic microscopes in earlier studies [38]. Using nucleosomes identified by NucleoMap, we predict the distribution of tetra-nucleosome structural motifs in hESC to investigate the relationship between tetra-nucleosome structural motifs and patterns in chromatin contact maps.

In our prediction task, binary classifiers are trained on the recently modeled yeast chromatin [24]. Because the spatial distances between nucleosome pairs are inversely proportional to some constant order of the contact frequency [39], 4-by-4 submatrices are extracted along the diagonal of the contact matrix as the input features. Ideally, chromatin contacts in the submatrices characterize the neighborhood of the nucleosomes. We observe in yeast that the number of contacts is closely related to the nucleosome structural motifs (S1 Table). In brief, *β*-rhombus tends to form neighborhoods with fewer contacts, while *α*-tetrahedron tends to form neighborhoods with more contacts. To improve the prediction accuracy, we divide the features into four groups according to the proportions of *α*-tetrahedrons with respect to the contact numbers (S3 Fig). Next, ten commonly used classifiers are trained and compared in each group respectively (S2 Table). At last, the models with the highest F1-scores in group2 (with 200–400 neighboring contacts), group3 (with 400–600 neighboring contacts), and group4 (with over 600 neighboring contacts) are selected and applied to hESC. Due to the overall low F1-scores, folding motifs of nucleosomes in group1 (with less than 200 neighboring contacts) are not predicted.

The ratio of predicted *α*-tetrahedron and *β*-rhombus in human (51.4% vs. 48.6%) are consistent with that in yeast (50.9% vs. 49.1%). We also observe that contact patterns in the neighborhood of *α*-tetrahedron and *β*-rhombus in human are similar to the patterns in yeast (S4 Fig). Together, these results imply that the classifiers trained on yeast successfully distinguish the folding motifs in human.

To investigate the correlation between the structural preference and genome function, we first compare the ratio of *α*-tetrahedron to *β*-rhombus at epigenetic marks, transcriptional factor binding sites, and candidate cis-regulatory element (cCRE) annotations. Surprisingly, almost all selected epigenetic marks and transcriptional factors exhibit a preference towards *α*-tetrahedron at their binding sites. On the contrary, the preferences on folding motifs vary among cis-regulatory elements. Three of the four cis-regulatory elements have certain preferences on the folding motifs. Higher levels of *α*-tetrahedron are observed at distal enhancers and enhancers, and more *β*-rhombus are observed at insulators. At promoters, the proportions of *α*-tetrahedron and *β*-rhombus are close to their global levels (Table 1). Combining the results from epigenetic marks, transcriptional factor binding sites, and cis-regulatory elements, it implies that although epigenetic marks and transcriptional factors bindings have a preference towards *α*-tetrahedrons, high-order genome functions still influence the final preference on tetra-nucleosome folding motifs.

**Table 1:** Preferences on tetra-nucleosome folding motifs in different regions.

Regions	Prop. of *α* motif	Prop. of *β* motif	Folding motif ratio	Preference	Significance
hESC chr21	51.4%	48.6%	1.00	NA	ns
**Epigenetic marks and transcriptional factor binding sites**					
CTCF	51.5%	48.5%	1.00	NA	ns
H3K27ac	51.9%	48.1%	1.02	*α*-tetrahedron	**
H3K36me3	51.7%	48.3%	1.01	*α*-tetrahedron	*
H3K4me1	53.2%	46.8%	1.12	*α*-tetrahedron	****
H3K4me2	52.6%	47.4%	1.05	*α*-tetrahedron	****
H3K4me3	51.8%	48.2%	1.02	*α*-tetrahedron	***
H3K79me2	51.6%	48.4%	1.00	NA	ns
H3K9ac	51.7%	48.3%	1.01	*α*-tetrahedron	**
H3K9me3	51.7%	48.3%	1.01	*α*-tetrahedron	*
H3K18ac	51.6%	48.4%	1.00	NA	ns
Nanog	51.9%	48.1%	1.02	*α*-tetrahedron	**
Rad21	51.6%	48.4%	1.00	NA	ns
H2AFZ	52.1%	47.9%	1.03	*α*-tetrahedron	**
GTF2F1	51.6%	48.4%	1.00	NA	ns
**cis-Regulatory elements**					
Distal enhancers	52.2%	47.8%	1.03	*α*-tetrahedron	****
Enhancers	54.5%	45.5%	1.14	*α*-tetrahedron	**
Promoters	50.9%	49.1%	0.98	*β*-rhombus	***
Insulators	46.3%	53.7%	0.813	*β*-rhombus	***
**Chromatin compartments**					
Compartment A	54.6%	45.4%	1.14	*α*-tetrahedron	****
Compartment B	46.8%	53.2%	0.83	*β*-rhombus	****
**SPIN states**					
Interior active1	58.5%	41.5%	1.34	*α*-tetrahedron	****
Interior active2	49.2%	50.8%	0.92	*β*-rhombus	****
Interior active3	43.9%	56.1%	0.74	*β*-rhombus	****
Interior repressive2	46.3%	53.7%	0.81	*β*-rhombus	****
Lamina	37.6%	62.4%	0.57	*β*-rhombus	****
Near lamina1	41.7%	58.3%	0.67	*β*-rhombus	****
Near lamina2	42.3%	57.7%	0.69	*β*-rhombus	****
Speckle	58.6%	41.4%	1.35	*α*-tetrahedron	****
**TAD boundaries**					
Strong boundaries	47.8%	25.2%	0.86	*β*-rhombus	****
Weak boundaries	69.2%	30.8%	2.12	*α*-tetrahedron	****
**Other chromatin structures**					
Loops	57.5%	42.5%	1.27	*α*-tetrahedron	****
Stripes	55.4%	44.6%	1.17	*α*-tetrahedron	****

Note: Folding motif ratio is calculated by comparing the *α*/*β* ratios in specific regions and the genome-wide *α*/*β* ratio. A folding motif ratio greater than 1 indicates that the region has a preference towards *α*-tetrahedrons, and a folding motif ratio smaller than 1 indicates a preference towards *β*-rhombus.

Meanwhile, the distribution of folding motifs also highly correlates with large-scale chromatin structures. We observe different proportions of *α*-tetrahedron and *β*-rhombus at multiple chromatin structures including compartments, topologically associated domain (TAD) boundaries, stripes, and loops. *α*-tetrahedrons present more frequently in compartment A (expression-active chromatin), while higher proportion of *β*-rhombuses is observed in compartment B (expression-inactive chromatin) (Table 1). At the level of nuclear subcompartments revealed by SPIN [40], we also observe consistent results. Among the eight identified SPIN-states, the highest proportions of *α*-tetrahedrons are found at two active states “Interior Active 1” (58.5%) and “Speckle” (58.6%), whereas the lowest proportions of *α*-tetrahedrons are found at inactive states “Lamina” (37.6%) and “Near Lamina 1” (37.6%) (Table 1). Moreover, we find that the preference for folding motifs changes at TAD boundaries according to the boundary strength. Rigid (“strong”) boundaries tend to form more *β*-rhombuses, and permissive (“weak”) boundaries tend to form more *α*-tetrahedrons (Table 1). Previous studies have shown that the strength of TAD boundaries is associated with their functionalities [41], possibly explaining the difference in their preferences on folding motifs. At loops and stripes, higher proportions of *α*-tetrahedrons are observed (Table 1). One possible explanation of the preference towards *α*-tetrahedron in these regions is that compacted local domains in chromatin contact maps, such as loop extrusion, play a role in the formation of compartment A [42].

### Nucleosome contact maps provide precise chromatin organizational details

Traditionally, ultra-high resolution contact maps are generated at certain fixed resolutions (e.g., 200bp). However, these 200bp-bins are not associated with genome structures in reality. As a result, studies of fine-scale nucleosome patterns such as zig-zag patterns are either limited in well-positioned regions (e.g., transcription factor binding sites) or using indirect statistics (e.g., contact profiles) [20, 22, 43]. To overcome this challenge, a nucleosome contact map, in which nodes represent actual nucleosomes, is generated in yeast to facilitate extraction and visualization of nucleosome motifs in previous studies [24]. In nucleosome contact maps, contacts assigned to nucleosome pairs are directly converted to edges between nodes, illustrating the spatial proximity between these nucleosomes. Using nucleosomes identified by NucleoMap, we generate nucleosome contact maps in multiple cell lines and compare them with two sets of related contact maps, including (1) 200bp-resolution contact maps and (2) nucleosome contact maps generated by iNucs [44], which generates nucleosome contact maps using pre-defined nucleosome positions and bin-based contact maps.

**Fig 4:**
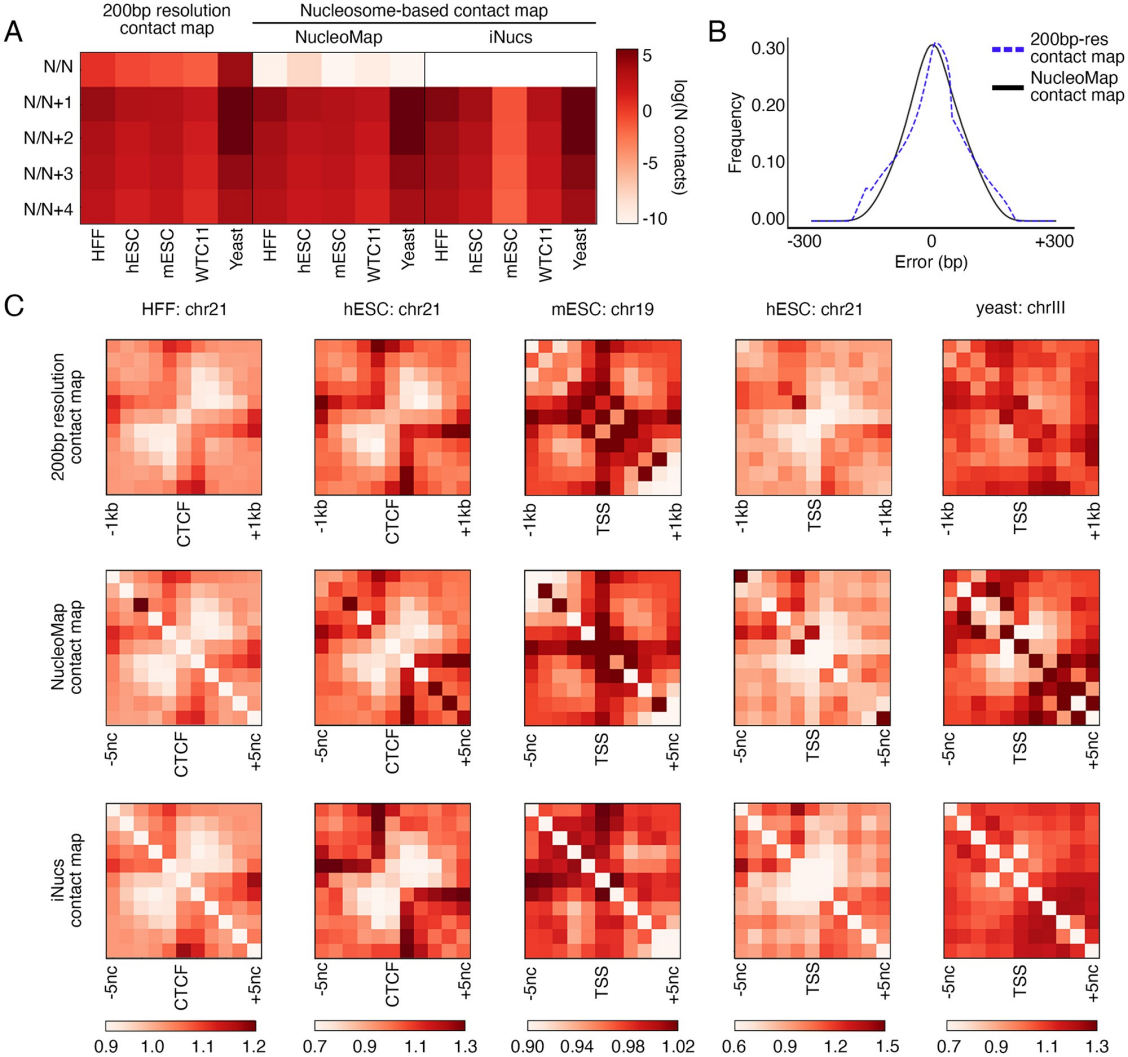
Nucleosome contact maps constructed by NucleoMap contain more concentrated inter-nucleosomal contact signals. A. Averaged contact numbers between neighboring nodes in 200bp-resolution contact maps and nucleosome contact maps constructed by NucleoMap and iNucs. Compared with 200bp-resolution contact maps, the number of self-contacts (N/N contacts) significantly decreases in nucleosome contact maps, which is more intuitive because the two ends of a contact connect different nucleosomes in a cell. B. Frequencies of contact distance errors after assigned to 200bp bins (blue) and nucleosomes identified by NucleoMap (black). The distance error of every contact is defined as the difference in contact distance after assigning both ends to their corresponding nodes in a chromatin contact map. Nucleosome contact maps achieve the same level of precision as 200bp-resolution contact maps. C. OE normalized pileup nucleosome contact maps and 200bp-resolution contact maps centered at CTCF binding sites or TSS regions in different cell lines. Nucleosome arrays are separated into two domains by CTCF binding sites and TSS flanking regions. Compared with 200bp-resolution contact maps, nucleosome contact maps reveal more concentrated patterns in most cell lines.

Compared with 200bp-resolution contact maps, nucleosome contact maps contain more interpretable and precise contact patterns. While having similar numbers of N/N+1, N/N+2, N/N+3, and N/N+4 contacts as 200bp-resolution contact maps, nucleosome contact maps generated by NucleoMap and iNucs barely include self contacts (N/N contacts), suggesting that most contacts connect two different nodes in nucleosome contact maps (Fig 4A). This property is consistent with the fact that every contact in the ultra-high resolution contact map consists of reads from two different nucleosomes. Although the contact distribution changes, nucleosome contact maps generated by NucleoMap still achieve the same level of precision as the commonly used 200bp-resolution bin-based contact maps, measured by the distance error between the aligned reads and their assigned node centers (Fig 4B). In addition, the error in nucleosome contact maps is symmetrically distributed compared with 200bp-resolution contact maps, because nucleosomes called by NucleoMap reflect the distribution of aligned reads, providing a more accurate presentation of the intrinsic inter-nucleosomal structures. Therefore, nucleosome contact maps capture the nucleosome organization within the nucleus more precisely than traditional bin-based chromatin contact maps.

Compared with 200bp-resolution bin-based contact maps, nucleosome contact maps better recover fine-scale nucleosomal structures. Nodes in traditional 200bp-resolution contact maps may not accurately cover the DNA wrapping around nucleosomes, and thus interactions between mono-nucleosomes are not precisely captured by the contacts between nodes. In contrast, contacts between two nodes in nucleosome contact maps intuitively represent the proximity of two nucleosomes. Since nucleosomes are basic structural components of chromatin, the maps better illustrate the fine-scale nucleosomal motifs. In the pileup maps centered at CTCF binding sites and TSSs in five cell lines, nucleosome contact maps provide more concentrated signals than 200bp-resolution bin-based contact maps (Fig 4C). In all pileup maps, nucleosome contact maps generated by NucleoMap and iNucs provide similar nucleosomal structures. A stronger contrast between the low contact frequency background and the high contact frequency looping structures anchored at CTCFs or TSSs is shown in the nucleosome contact maps, allowing easier identification of spatial nucleosome motifs.

## Discussion

Incorporating inter-nucleosome distance information reveals more detailed and precise nucleosome positioning throughout the genome. Here we report a computational approach, NucleoMap, for nucleosome identification in both well-positioned and poorly-positioned regions from ultra-high resolution contact maps. Using public Micro-C data from yeast, human, and mouse, we demonstrated that NucleoMap effectively detects nucleosomes in complex mammalian genomes, where most nucleosomes are poorly positioned. Using an ablation experiment, we justify that all factors in NucleoMap (i.e., the aligned reads, the binding preference, and the pairing information) contribute to the final results (S7 Fig). As the resolution of 3D chromatin organization profiling reaches the nucleosome level, nucleosome contact maps present more precise inter-nucleosome contact patterns than classical fixed-bin resolution contact maps.

The genome-wide nucleosome positioning identified by NucleoMap provides an opportunity to revisit epigenetic mark data at mono-nucleosome resolution. Although it has long been known that epigenetic marks are decorated on mono-nucleosomes, previous studies rarely explore the mono-nucleosome level due to the ubiquitously distributed poorly-positioned nucleosomes in complex eukaryote genomes. The genome-wide nucleosome map enhances existing epigenetic mark data to mono-nucleosome level, especially in the poorly positioned transcriptionally silent chromatin. Furthermore, by integrating epigenetic modifications and properties of nucleosome arrays in different genome regions, it is possible to establish a more comprehensive understanding of gene regulation. However, we note that more accurate mapping of epigenetic signals to mono-nucleosomes requires both ultra-high resolution epigenetic signals such as CUT&RUN data and enhanced computational approaches that consider the densities of mono-nucleosomes and epigenetic signals. Follow-up work is still required to design computational methods specifically for mono-nucleosome level sequencing data mapping.

The produced nucleosome contact maps allow a comprehensive analysis of the association between nucleosome spatial organization and genome functions. Using nucleosome contact maps, it is possible to extract and locate nucleosome folding patterns across the genome. Although some computational approach has been developed to model inter-nucleosomal contacts [44], NucleoMap is the first method jointly identifying nucleosomes and modeling inter-nucleosomal contacts. Using the nucleosome contact map constructed by NucleoMap, the hierarchical chromatin structures such as tetra-nucleosome folding motifs are retrieved by *in silico* approaches in the human genome. Although the exact spatial constructions of *α*-tetrahedron and *β*-rhombus are still under discussion [45], the existence of tetra-nucleosome folding motifs is confirmed in Cryo-EM studies [38], providing an experimental foundation of studying second-order nucleosome folding motifs in human genome via ultra-high resolution contact maps. It is possible to identify more accurate tetra-nucleosome folding motifs and higher-level chromatin folding structures with the help of enhanced machine learning models that utilize the sequential nature of the chromatin. Furthermore, combined with the epigenetic signals annotated to mono-nucleosomes, it is also possible to establish a 3D framework illustrating the spatial structures of epigenetic events. Compared with traditional studies in this area which focus on the interactions along the linear DNA sequence, this framework unveils interactions of chromatin modifications in an ultra-high resolution 3D space, and thus provides additional knowledge in the regulation of genome activities.

## Methods

### NucleoMap algorithms

#### Estimating read centers in Micro-C contact maps

To estimate the true read density along the genome, we first estimate positions of read centers, which are not directly accessible from the alignment data. Each contact in a chromatin contact map is composed of two anchor reads, with various sizes from ∼120bp to ∼170bp each, referring to two different nucleosomes (S5 Fig step 4). During the paired-end sequencing and downstream processing, only ∼50bp fragments at two ends of a contact are sequenced and mapped to the reference genome, and thus the centers of the two anchor reads are not sequenced and mapped in the alignment data (S5 Fig step 6). Therefore, read centers need to be estimated from the mapped fragments.

One effective way to estimate read centers is to shift the ends towards 3’ direction by half of the average read size. NucleoMap automatically estimates the average read size in the Micro-C data using the difference in contact distances across contact types. Four types of contacts, ++, +−, −+, and −−, can be found in the chromatin contact map, according to the strands the reads mapped to (S6 Fig). Contacts of different types vary in contact distance even when they anchor the same nucleosome pair. +− contacts cover two nucleosome dyads and the fragment between them, and ++ contacts and −− contacts cover a nucleosome dyad and the fragment between them, while −+ contacts cover only the fragment between them. Based on this observation, the average read size is calculated as the average difference in contact distances between +− contacts and ++ contacts with three steps. First, NucleoMap calculates the contact distance distributions of short-range +− and ++ contacts. Next, peaks are called from the two distributions. The first peak centers in the histograms correspond to the average contact distance between neighboring nucleosomes in +− and ++ contacts. Finally, the average read size is estimated to be the distance between the first peak centers in the two distributions.

After shifting reads to their centers, both reads in the contacts with genomic distances shorter than 160bp are excluded in the downstream analysis to prevent artifacts introduced by the outliers.

#### Calculating sequence-based binding score

Sequence-based binding score measures sequence-based nucleosome affinity at a given position. The score is calculated by normalizing the convolution score of an AA/AT/TA/TT dinucleotide PWM. The binding score is calculated in four steps. First, NucleoMap calculates a Position Frequency Matrix (PFM) of AA/AT/TA/TT dinucleotides. PFM records the occurrences of AA/AT/TA/TT dinucleotides at each position within ±80bp from the *N* read centers. Based on the dinucleotides frequency, a 2 × 160 PFM *F* is generated by

$$\begin{array}{c} F_{k,j}=\frac{1}{N}\sum_{i=1}^{N}\delta \left(X_{i,j}=k\right), \end{array}$$ (1)

where *i* ∈ [1, *N*], *j* ∈ [1, 200] and *k* = {0, 1} indicates the occurrence of AA/AT/TA/TT dinucleotides. *δ* is an indicator function. The first row of the matrix indicates the occurring frequency of AA/AT/TA/TT dinucleotides, and the second row indicates the occurring frequency of other dinucleotides. Following that, a PWM *W* expressing the binding patterns is calculated as

$$\begin{array}{c} W_{k,j}={\text{log}}_{2}\left(F_{k,j}/b_{k}\right), \end{array}$$ (2)

where *b*_*k*_ denotes the background frequencies of AA/AT/TA/TT dinucleotides and other dinucleotides calculated from the reference genome. In the third step, NucleoMap calculates the cross correlation scores *D* between the PWM and the one-hot encoded reference genome.

$$\begin{array}{cc} & D\left(i\right)=\sum_{k,j}W_{k,j}G\left(i\right), \end{array}$$ (3)

where *G*(*i*) is the dinucleotide in reference genome at position *i*. This score illustrates the similarity between the nucleosome binding pattern and the genome sequence at a given position. In the last step, a binding score *B* is generated by normalizing the cross correlation score *D* in two steps. First, $\tilde{B}(i)$ is generated by normalizing *D* over its neighborhood,

$$\begin{array}{c} \tilde{B}\left(i\right)=\frac{D(i)}{\sum_{j=i-50}^{i+50}D\left(j\right)}, \end{array}$$ (4)

Next, *B*(*i*) is calculated by *z*-normalizing $\tilde{B}(i)$,

$$\begin{array}{c} B\left(i\right)=\frac{\tilde{B}(i)-E(\tilde{B})}{\sigma (\tilde{B})}. \end{array}$$ (5)

The resulting binding score quantifies in nucleosome binding preference at a position compared with its neighborhood.

#### Estimating nucleosome numbers and defining the objective function

Reads are assigned to nucleosomes within 1kb using a hard clustering DP mixture model with a fixed covariance *σ*^2^ *I*. We assume that within 1kb on the genome, reads *X* = {*x*_*i*_} are samples drawn from an unknown number of Gaussian distributions with fixed covariance *σ*^2^, representing the nucleosome dyad. Under this assumption, a DP mixture model of nucleosomes is formulated as follows:

$$x_{i}∼N(\mu_{c},\sigma^{2}I),$$ (6)

$$\mu_{c}∼G,$$ (7)

$$G∼\text{DP}(\alpha ,G_{0}).$$ (8)

Here $G_{0}=N\left(0,I\right)$ is a prior over the mean distributions of the Gaussian mixtures, and a draw $G=\sum_{c=1}^{\infty }\pi_{c}\delta \left(\mu_{c}\right)$ from *G*_0_ is the mean distribution of a Gaussian mixture, where *π*_*c*_ denotes the weight of the *c*-th Gaussian component. For *i* = 1, 2, …, *n*, the probability *p*_*c*_ of assigning a read *x*_*i*_ to an existing nucleosome *c* is

$$\begin{array}{c} p_{c}=\frac{n_{c}\cdot \text{exp}(-\frac{1}{2\sigma }d_{c}^{2})}{\text{exp}(-\frac{1}{2\sigma }[\lambda +\frac{\sigma }{1+\sigma }d_{0}^{2}])+\sum_{j=1}^{k}n_{j}\cdot \text{exp}(-\frac{1}{2\sigma }d_{j}^{2})}, \end{array}$$ (9)

where *n*_*c*_ is the number of reads assigned to nucleosome *c*, *d*_0_ = ‖*x*_*i*_‖, *d*_*c*_ = ‖*x*_*i*_ − *μ*_*c*_‖, and $\lambda =-2\sigma \text{ln}({\left(1+\frac{1}{\sigma }\right)}^{1/2}\alpha )$. Similarly, the read *x*_*i*_ is assigned to a new nucleosome with a probability *p*_*new*_

$$\begin{array}{c} p_{new}=\frac{\text{exp}(-\frac{1}{2\sigma }[\lambda +\frac{\sigma }{1+\sigma }d_{0}^{2}])}{\text{exp}(-\frac{1}{2\sigma }[\lambda +\frac{\sigma }{1+\sigma }d_{0}^{2}])+\sum_{j=1}^{k}n_{j}\cdot \text{exp}(-\frac{1}{2\sigma }d_{j}^{2})}. \end{array}$$ (10)

A hard assignment DP mixture model is obtained by pushing *σ* → 0. When *σ* approaches 0, the numerator of *p*_*new*_ is dominated by λ. Furthermore, as *σ* → 0, the assignment probabilities become binary and only the smallest values of $\left\{d_{1}^{2},d_{2}^{2},\ldots ,d_{k}^{2},\lambda \right\}$ receive a non-zero probability. In particular, a new nucleosome is created whenever a read is farther than $\sqrt{\lambda }$ bp away from every existing nucleosome center. The underlying objective of this model is similar to the *k*-means objective function,

$$\begin{array}{c} \underset{{\left\{\ell_{c}\right\}}_{c=1}^{k}}{\text{min}}\;\sum_{c=1}^{k}\sum_{x\in \ell_{c}}d_{c}^{2}+\lambda k, \end{array}$$ (11)

where *ℓ*_*c*_ is the set of reads assigned to nucleosome *c*. The threshold λ controls the trade-off between the traditional *k*-means term and the cluster penalty term. Optimizing this objective function identifies potential nucleosome centers based on the read density.

#### Integrating read density, pairing information, and binding scores

To incorporate pairing information and binding scores, an adjusted distance ${\tilde{d}}_{c}$ is used instead of *d*_*c*_. We define

$$\begin{array}{c} {\tilde{d}}_{c}\left(x_{i}\right)=d_{c}+\gamma_{1}\delta_{c}\left(x_{i}^{\prime }\right)+\gamma_{2}B\left(\mu_{c}\right), \end{array}$$ (12)

where $x_{i}^{\prime }$ is the other read sharing a contact with *x*_*i*_, *δ*_*c*_ an indicator function returning 1 if $x_{i}^{\prime }\in \ell_{c}$ and 0 otherwise,, *B*(*μ*_*c*_) the binding score of predicted nucleosome center, and *γ*_1_, *γ*_2_ the corresponding distance penalties. The final objective function is

$$\begin{array}{c} \underset{{\left\{\ell_{c}\right\}}_{c=1}^{k}}{\text{min}}\;\sum_{c=1}^{k}\sum_{x\in \ell_{c}}{{\tilde{d}}_{c}}^{2}+\lambda k. \end{array}$$ (13)

The model is optimized using a previously proposed hard clustering algorithm that behaves similarly to *k*-means with the exception that new clusters are formed when the aforementioned condition is satisfied [46].

### Identifying nucleosomes from Micro-C data

Alignment files of Micro-C data are downloaded from 4DN data portal (human cell lines), or generated by Bowtie2 with ‘very sensitive’ mode (mESC and yeast) using reference genomes hg38, mm10, and SacCer3 respectively [47]. Mapped reads from all replicates are merged before calling nucleosomes. Using the alignment files and the following parameters, we benchmarked NucleoMap and multiple baseline methods including DANPOS2 [10], NOrMAL [11], Nseq [13], and nucleR [29]. NucleoMap is run with default parameters. DANPOS2 is run with parameters “-m 0 -p 0.05”. NOrMAL is run using the original config.txt on its GitHub repository. Nseq is run with parameters “-f 0.01 -s 10 -t 16”. nucleR is run with parameters “threshold = “25%”, score = TRUE, width = 147”.

### Calculating nucleosome occupancy

Nucleosome occupancy measures the fraction of nucleosomes covering a given position in a cell population. The measure is originally proposed by Valouev et al. [18] to describe the nucleosome positioning level, but here a smaller neighborhood *w* = 30 is chosen in the normalization step to increase its detection sensitivity.

The nucleosome occupancy is calculated in three steps. First, a smoothing kernel *K* is defined as

$$\begin{array}{c} K\left(i,w\right)={\left(1-{\left(i/w\right)}^{2}\right)}^{3}\delta \left(|i|<w\right), \end{array}$$ (14)

where *w* defines an aggregation window and *δ* is an indicator function. In the second step, we generate the read coverage files from the alignment files using samtools depth with parameters “-a -H -Q 10” [48]. Next, the convolution kernel *K* is applied to the read coverage file along the chromatin

$$\begin{array}{c} D\left(i,w=30\right)=\sum_{j=0}^{L}K\left(i,w\right)d\left(j\right), \end{array}$$ (15)

where *L* is the length of the chromatin and *d*(*j*) represents the number of read centers at position *j*. At last, the smoothed density is normalized over its neighborhood

$$\begin{array}{c} S\left(i,w=30\right)=\frac{D(i,w)}{\sum_{j=i-4*w}^{i+4*w}\frac{1.09}{w}D\left(j,w\right)}. \end{array}$$ (16)

A scaling factor 1.09 is designed to normalize the occupancy values as

$$\begin{array}{c} \int_{-1}^{1}{\left(1-u^{2}\right)}^{3}du=1/1.09. \end{array}$$ (17)

The neighborhood size in the denominator is set to ±4 * *w* such that it covers a slightly larger region than a well-positioned nucleosome (146bp) to capture the poorly positioned nucleosomes.

### Annotating genome features to mono-nucleosomes

Epigenetic modification peaks are assigned to the nearest nucleosomes to the peak centers. In this way, we generate binarized signals indicating whether or not a nucleosome is subjected to certain modifications, and peak strengths and fold changes are ignored. Similarly, mono-nucleosome positioning levels are calculated using the nucleosome occupancy signal. We define the highest occupancy value within ± 30bp from a nucleosome center as its normalized positioning level. Cis-regulatory elements are annotated to all nucleosomes within a ± 500bp neighborhood.

Nucleosomes within the span of compartments, SPIN states, or stripes are assigned with the corresponding features. TAD boundaries are annotated to all nucleosomes within a ± 500bp neighborhood. Loops are annotated to all nucleosomes within a ± 500bp neighborhood at each anchor.

### Predicting tetra-nucleosome folding motifs

For the *i*-th nucleosome, we generate a 10-dimension feature using elements from the upper triangle of the sub-contact-matrix containing the (*i* − 1)-th, the *i*-th, the (*i* + 1)-th, and the (*i* + 2)-th nucleosomes. Tetra-nucleosome motif labels of the yeast genome are collected from the nucleosome 3D coordinates generated in a published study [24].

Nucleosomes are grouped according to the sum of their features. Ten classifiers from the sklearn python package are trained in each group, including k-Nearest Neighbors, Linear SVM, RBF SVM, Gaussian Process, Decision Tree, Random Forest, Multilayer Perceptron, AdaBoost, Gaussian Naive Bayes, and Quadratic Discriminant Analysis. The parameters in these models are as follow: KNeighborsClassifier(k = 3), SVC(kernel = “linear”, C = 0.025), SVC(gamma = 2, C = 1), GaussianProcessClassifier(1.0 * RBF(1.0)), DecisionTreeClassifier(max_depth = 5), RandomForestClassifier(max_depth = 5, n_estimators = 10, max_features = 1), MLPClassifier(alpha = 1, max_iter = 1000), AdaBoostClassifier(), GaussianNB(), and QuadraticDiscriminantAnalysis().

In each group, 75% of the nucleosomes are randomly selected as training data, and the remaining nucleosomes are used as test set. The classifiers are trained on the training data, and their performances are evaluated by F1-scores on the test set.

The folding motif preference is measured by a folding motif ratio within a specific region, defined as

$$\begin{array}{c} \frac{N_{\text{local}}^{\left(\alpha \right)}\times N_{\text{genome}}^{\left(\beta \right)}}{N_{\text{local}}^{\left(\beta \right)}\times N_{\text{genome}}^{\left(\alpha \right)}}. \end{array}$$ (18)

When this ratio > 1, the region has a preference towards *α*-tetrahedron and towards *β*-rhombus otherwise. The significance of folding motif preferences is evaluated using two-sided *T*-tests. Folding motif ratios are compared between nucleosomes within specific regions and nucleosomes sampled from the whole genome.

### Constructing nucleosome contact maps and OE normalization

Nucleosome contact maps are constructed by assigning contacts to their corresponding nucleosomes identified by NucleoMap. NucleoMap estimates the expected contact numbers between nucleosome pairs according to their genomic distance. Based on the assumption that the contact frequency is a function of genomic distance, the expected contact numbers between two nucleosomes is estimated given their genomic distance *d*,

$$\begin{array}{c} C_{\text{exp}}\left(d\right)=\frac{N_{\text{c}}\left(d\right)}{NP_{\text{nuc}}\left(d\right)}, \end{array}$$ (19)

where *N*_c_ refers to the total number of contacts in the contact map with genomic distance *d*, and *NP*_nuc_ refers to the total number of nucleosome pairs in the contact map with genomic distance *d*.

However, it is difficult to calculate *NP*_nuc_ directly in practice because it requires a computational complexity of *O*(*n*^2^), where *n* is the number of nucleosomes in the contact map. To avoid the expensive computation, we instead estimate *NP*_nuc_ with a summation over multiple Erlang distributions. Assuming that nucleosomes occur at a steady rate along the genome, the genomic distances between neighboring nucleosomes follow an exponential distribution

$$\begin{array}{c} f\left(d;\lambda \right)=\lambda e^{-\lambda d}, \end{array}$$ (20)

where *d* is the genomic distance and λ = *L*_chrom_/*N*_nuc_ is the occurring rate of nucleosomes. Therefore, the genomic distance between the *i*-th and the (*i* + *k*)-th nucleosomes follow an Erlang distribution which characterizes the sum of *k* independent exponential distributions

$$\begin{array}{c} g\left(d;k,\lambda \right)=\frac{\lambda^{k}d^{k-1}e^{-\lambda d}}{(k-1)!}. \end{array}$$ (21)

Hence the probability of having *k* nucleosomes within a certain range of genomic distance [*d*_1_, *d*_2_], denoted by *P*(*d*_1_, *d*_2_; *k*, λ), is calculated by the difference in CDF of the Erlang distribution,

$$\begin{array}{c} P\left(d_{1},d_{2};k,\lambda \right)=\left(N_{\text{nuc}}-k\right)\times \sum_{i=1}^{k-1}\frac{1}{n!}(e^{-\lambda d_{2}}{\left(\lambda d_{2}\right)}^{n}-e^{-\lambda d_{1}}{\left(\lambda d_{1}\right)}^{n}), \end{array}$$ (22)

and the expected number of nucleosome pairs within the range [*d*_1_, *d*_2_] in the chromatin, denoted by *NP*_nuc_(*d*_1_, *d*_2_), is calculated by summing the differences of multiple Erlang distributions under a series of *k*s,

$$\begin{array}{c} NP_{\text{nuc}}\left(d_{1},d_{2}\right)=\sum_{k=1}^{N_{\text{nuc}}}P\left(d_{1},d_{2};k,\lambda \right). \end{array}$$ (23)

To further reduce computational complexity, this number is approximated by a smaller set of *k*s

$$\begin{array}{c} NP_{\text{nuc}}\left(d_{1},d_{2}\right)\approx \sum_{k\in s}P\left(d_{1},d_{2};k,\lambda \right), \end{array}$$ (24)

where max(1, *d*_1_/150 − 20) ≤ *s* ≤ min(*N*_nuc_, *d*_2_/150 + 20). The OE normalized contacts between two nucleosomes are finally given by the ratio between observed contacts and the expected contacts,

$$\begin{array}{c} C_{\text{OE}}\left(d\right)=\frac{C_{\text{obs}}}{C_{\text{exp}}\left(d\right)}, \end{array}$$ (25)

where *C*_obs_ is the contact numbers between the nucleosome pairs, and *d* is their genomic distance.

## Data access

Data and the source code in this paper are publicly accessible (Table 2). Majority of the sequencing data involved in the this paper are public available in NCBI GEO repository, ENCODE project, and 4DN data portal. Software used in this paper is available on GitHub. A python implementation of NucleoMap is provided on GitHub, which takes processed contact pair files as input and generates nucleosome contact maps. Loops in hESC micro-C data are called with Juicer HiCCUPS algorithm. Stripes in hESC micro-C data are called by the stripe caller developed by our group. SPIN state data are from a published study.

**Table 2:** Data and softwares involved in this paper.

Resource	Source	Identifier
**Ultra-high resolution contact maps**		
hESC Micro-C	4DN data portal	4DNES21D8SP8
HFF Micro-C	4DN data portal	4DNESWST3UBH
WTC11 Micro-C	4DN data portal	4DNESODGV2V2
mESC Micro-C	NCBI GEO repository	GSE130275
yeast Micro-C	NCBI GEO repository	GSE68016
**Gene expression profile**		
hESC	ENCODE project	ENCFF038OTF
WTC11	NCBI GEO repository	GSE139273
**Epigenetic signals**		
H3K4ac	ENCODE project	ENCFF604GSC
H3K9ac	ENCODE project	ENCFF719SGF
H3K27ac	ENCODE project	ENCFF162HPV
H3K4me1	ENCODE project	ENCFF238YJA
H3K4me2	ENCODE project	ENCFF583ABZ
H3K4me3	ENCODE project	ENCFF456NIF
H3K9me3	ENCODE project	ENCFF654ZZO
H3K27me3	ENCODE project	ENCFF254ACI
H3K36me3	ENCODE project	ENCFF813VFV
H3K79me1	ENCODE project	ENCFF088PTH
H3K79me2	ENCODE project	ENCFF620GIW
H4K8ac	ENCODE project	ENCFF760EFQ
H4K20me1	ENCODE project	ENCFF718VCC
H2AFZ	ENCODE project	ENCFF584JOM
POLR2A	ENCODE project	ENCFF322DAE
CTCF	ENCODE project	ENCFF368LWM
RAD21	ENCODE project	ENCFF532ZYE
**Chromatin segmentation**		
25-state ChromHMM segmentations	ENCODE project	ENCSR604YKJ
**Structural annotation**		
AB compartments	4DN data portal	4DNFI475YIT8
TAD boundaries	4DN data portal	4DNFIED5HLDC
SPIN states	NCBI GEO repository	GSE148362; GSE148609
**candidate cis-regulatory element**		
candidate cis-regulatory element	ENCODE project	ENCSR597SZL
**Software and algorithms**		
Stripe caller	GitHub	https://github.com/dmcbffeng/StripeCaller
DANPOS-2.2.2	GitHub	https://github.com/sklasfeld/DANPOS3
nucleR	Bioconductor	https://github.com/nucleosome-dynamics/nucleR
NOrMAL	GitHub	https://github.com/antonpolishko/NOrMAL
Nseq	GitHub	https://github.com/songlab/NSeq
NucleoMap	This paper	https://github.com/liu-bioinfo-lab/NucleoMap

## Supporting information

S1 Fig Dinucleotide PWMs and the resulting binding scores. A. Dinucleotide PWMs of yeast (left) and hESC (right). The dinucleotide PWMs in yeast and hESC reflect similar nucleosome binding preference of ∼10bp periodic AA/AT/TA/TT 2-mers in the two cell lines. B. Average nucleosome-binding scores around experimentally identified nucleosomes (left) and computational identified nucleosomes (right) in yeast. Peaks of motif-based nucleosome-binding scores centered at both experimentally and computationally identified nucleosomes indicate that the nucleosome-binding score defined in NucleoMap effectively captures the nucleosome sequence preference.
(TIF)

S2 Fig Average contact distance characterizes local nucleosome spatial organization. Frequencies of inter-nucleosome contacts correlate with the spatial distance between nucleosome pairs. In tightly packed chromatin, the neighborhoods of central nucleosomes involve more adjacent nucleosomes (±3 nucleosomes in the example) and thus forming longer average contact distances, whereas in lightly packed chromatin, fewer nucleosomes are involved in the neighborhoods of the central nucleosomes, forming shorter average contact distances.
(TIF)

S3 Fig Percentages of *α*-tetrahedrons against local contact numbers. Neighborhood with more contacts tends to have higher percentages of *α*-tetrahedrons. The nucleosomes are divided into four groups according to their local contact numbers. Within each group, the slope (the trend of forming *α*-tetrahedrons with respect to contact numbers) is approximately constant.
(TIF)

S4 Fig Average local contact maps of the two tetra-nucleosome folding motifs. Nucleosomes of *α*-tetrahedrons and *β*-rhombuses predicted by machine learning models in human embryonic stem cells have consistent local contact patterns with yeast. Here average local contact maps of the two tetra-nucleosome motifs between the *i*−1-th and the *i*+2-th nucleosomes are presented. Values in the contact maps are OE normalized.
(TIF)

S5 Fig Read centers are not directly accessible in Micro-C libraries. The Micro-C libraries are generated as follows. 1. Fix chromatin with formaldehyde. 2. Digest linker DNA in crosslinked chromatin with MNase. In this step, MNase does not strictly digest linker DNA. A small fraction of linker DNA is remained, while core DNA in some nucleosomes is partially digested. 3. Ligate the ends of remaining DNA with biotin according to their spatial proximity. 4. Digest protein and extract ligated DNA contacts. 5. Pair-end sequencing of the contacts. 6. Micro-C libraries are generated, containing the ∼50bp sequence of one end of every nucleosome.
(TIF)

S6 Fig Four types of inter-nucleosome contacts vary in contact distance. Biotin ligation is formed between the closest ends of core DNAs wrapping around the nucleosome pairs. Depending on the nucleosome orientation, four types of contacts can be formed between two nucleosomes, namely, +−, −−, ++, and −+. Even if they anchor the same nucleosome pairs (e.g., contacts between N/N+1 nucleosomes), different contact types vary in contact distance measured by the genomic distance between two ends of a contact.
(TIF)

S7 Fig Contribution of different factors in NucleoMap measured by recall (left) and precision (right). The aligned reads play the most crucial role in detecting nucleosomes, accounting for the largest areas under the curves for both precision and recall. The pairing information significantly improves the recall of NucleoMap, and it also contributes to the precision of our method. The binding preferences improve the precision and recall when *d*_*t*_ is small, suggesting that it helps locat nucleosomes more accurately.
(TIF)

S1 Table Proportions of tetra-nucleosome motifs in different groups in yeast. (XLSX)

S2 Table F1-scores of different folding motif classifiers. (XLSX)

## References

[1] Misteli T. Beyond the sequence: cellular organization of genome function. Cell. 2007;128(4):787–800. doi: 10.1016/j.cell.2007.01.028 17320514

[2] Fletcher TM, Hansen JC. The nucleosomal array: structure/function relationships. Critical Reviews in Eukaryotic Gene Expression. 1996;6(2-3). doi: 10.1615/CritRevEukarGeneExpr.v6.i2-3.40 8855387

[3] Yuan GC, Liu YJ, Dion MF, Slack MD, Wu LF, Altschuler SJ, et al. Genome-scale identification of nucleosome positions in S. cerevisiae. Science. 2005;309(5734):626–630. doi: 10.1126/science.1112178 15961632

[4] Zhang T, Zhang W, Jiang J. Genome-wide nucleosome occupancy and positioning and their impact on gene expression and evolution in plants. Plant physiology. 2015;168(4):1406–1416. doi: 10.1104/pp.15.00125 26143253 PMC4528733

[5] Lai B, Gao W, Cui K, Xie W, Tang Q, Jin W, et al. Principles of nucleosome organization revealed by single-cell micrococcal nuclease sequencing. Nature. 2018;562(7726):281–285. doi: 10.1038/s41586-018-0567-3 30258225 PMC8353605

[6] Baldi S, Krebs S, Blum H, Becker PB. Genome-wide measurement of local nucleosome array regularity and spacing by nanopore sequencing. Nature structural & molecular biology. 2018;25(9):894–901. doi: 10.1038/s41594-018-0110-0 30127356

[7] Johnson SM, Tan FJ, McCullough HL, Riordan DP, Fire AZ. Flexibility and constraint in the nucleosome core landscape of Caenorhabditis elegans chromatin. Genome research. 2006;16(12):1505–1516. doi: 10.1101/gr.5560806 17038564 PMC1665634

[8] Song L, Crawford GE. DNase-seq: a high-resolution technique for mapping active gene regulatory elements across the genome from mammalian cells. Cold Spring Harbor Protocols. 2010;2010(2):pdb–prot5384. doi: 10.1101/pdb.prot5384 20150147 PMC3627383

[9] Buenrostro JD, Wu B, Chang HY, Greenleaf WJ. ATAC-seq: a method for assaying chromatin accessibility genome-wide. Current protocols in molecular biology. 2015;109(1):21–29. doi: 10.1002/0471142727.mb2129s109 25559105 PMC4374986

[10] Chen K, Xi Y, Pan X, Li Z, Kaestner K, Tyler J, et al. DANPOS: dynamic analysis of nucleosome position and occupancy by sequencing. Genome research. 2013;23(2):341–351. doi: 10.1101/gr.142067.112 23193179 PMC3561875

[11] Polishko A, Ponts N, Le Roch KG, Lonardi S. NORMAL: accurate nucleosome positioning using a modified Gaussian mixture model. Bioinformatics. 2012;28(12):i242–i249. doi: 10.1093/bioinformatics/bts206 22689767 PMC3371838

[12] Chen W, Liu Y, Zhu S, Green CD, Wei G, Han JDJ. Improved nucleosome-positioning algorithm iNPS for accurate nucleosome positioning from sequencing data. Nature communications. 2014;5(1):1–14. 25233085 10.1038/ncomms5909

[13] Nellore A, Bobkov K, Howe E, Pankov A, Diaz A, Song JS. NSeq: a multithreaded Java application for finding positioned nucleosomes from sequencing data. Frontiers in genetics. 2013;3:320. doi: 10.3389/fgene.2012.00320 23335939 PMC3542818

[14] Mammana A, Vingron M, Chung HR. Inferring nucleosome positions with their histone mark annotation from ChIP data. Bioinformatics. 2013;29(20):2547–2554. doi: 10.1093/bioinformatics/btt449 23981350 PMC3789549

[15] Vainshtein Y, Rippe K, Teif VB. NucTools: analysis of chromatin feature occupancy profiles from high-throughput sequencing data. BMC genomics. 2017;18(1):1–13. doi: 10.1186/s12864-017-3580-2 28196481 PMC5309995

[16] Schep AN, Buenrostro JD, Denny SK, Schwartz K, Sherlock G, Greenleaf WJ. Structured nucleosome fingerprints enable high-resolution mapping of chromatin architecture within regulatory regions. Genome research. 2015;25(11):1757–1770. doi: 10.1101/gr.192294.115 26314830 PMC4617971

[17] Tarbell ED, Liu T. HMMRATAC: a Hidden Markov ModeleR for ATAC-seq. Nucleic acids research. 2019;47(16):e91–e91. doi: 10.1093/nar/gkz533 31199868 PMC6895260

[18] Valouev A, Johnson SM, Boyd SD, Smith CL, Fire AZ, Sidow A. Determinants of nucleosome organization in primary human cells. Nature. 2011;474(7352):516–520. doi: 10.1038/nature10002 21602827 PMC3212987

[19] Beshnova DA, Cherstvy AG, Vainshtein Y, Teif VB. Regulation of the nucleosome repeat length in vivo by the DNA sequence, protein concentrations and long-range interactions. PLoS computational biology. 2014;10(7):e1003698. doi: 10.1371/journal.pcbi.1003698 24992723 PMC4081033

[20] Hsieh THS, Weiner A, Lajoie B, Dekker J, Friedman N, Rando OJ. Mapping nucleosome resolution chromosome folding in yeast by micro-C. Cell. 2015;162(1):108–119. doi: 10.1016/j.cell.2015.05.048 26119342 PMC4509605

[21] Hsieh THS, Fudenberg G, Goloborodko A, Rando OJ. Micro-C XL: assaying chromosome conformation from the nucleosome to the entire genome. Nature methods. 2016;13(12):1009–1011. doi: 10.1038/nmeth.4025 27723753

[22] Hsieh THS, Cattoglio C, Slobodyanyuk E, Hansen AS, Rando OJ, Tjian R, et al. Resolving the 3D landscape of transcription-linked mammalian chromatin folding. Molecular cell. 2020;78(3):539–553. doi: 10.1016/j.molcel.2020.03.002 32213323 PMC7703524

[23] Ramani V, Cusanovich DA, Hause RJ, Ma W, Qiu R, Deng X, et al. Mapping 3D genome architecture through in situ DNase Hi-C. Nature protocols. 2016;11(11):2104–2121. doi: 10.1038/nprot.2016.126 27685100 PMC5547819

[24] Ohno M, Ando T, Priest DG, Kumar V, Yoshida Y, Taniguchi Y. Sub-nucleosomal genome structure reveals distinct nucleosome folding motifs. Cell. 2019;176(3):520–534. doi: 10.1016/j.cell.2018.12.014 30661750

[25] Wagstaff K, Cardie C, Rogers S, Schrödl S, et al. Constrained k-means clustering with background knowledge. In: Icml. vol. 1; 2001. p. 577–584.

[26] Segal E, Fondufe-Mittendorf Y, Chen L, Thåström A, Field Y, Moore IK, et al. A genomic code for nucleosome positioning. Nature. 2006;442(7104):772–778. doi: 10.1038/nature04979 16862119 PMC2623244

[27] Reynolds SM, Bilmes JA, Noble WS. Learning a weighted sequence model of the nucleosome core and linker yields more accurate predictions in Saccharomyces cerevisiae and Homo sapiens. PLoS computational biology. 2010;6(7):e1000834. doi: 10.1371/journal.pcbi.1000834 20628623 PMC2900294

[28] Brogaard K, Xi L, Wang JP, Widom J. A map of nucleosome positions in yeast at base-pair resolution. Nature. 2012;486(7404):496–501. doi: 10.1038/nature11142 22722846 PMC3786739

[29] Flores O, Orozco M. nucleR: a package for non-parametric nucleosome positioning. Bioinformatics. 2011;27(15):2149–2150. doi: 10.1093/bioinformatics/btr345 21653521

[30] Jiang C, Pugh BF. Nucleosome positioning and gene regulation: advances through genomics. Nature Reviews Genetics. 2009;10(3):161–172. doi: 10.1038/nrg2522 19204718 PMC4860946

[31] Gaffney DJ, McVicker G, Pai AA, Fondufe-Mittendorf YN, Lewellen N, Michelini K, et al. Controls of nucleosome positioning in the human genome. PLoS genetics. 2012;8(11):e1003036. doi: 10.1371/journal.pgen.1003036 23166509 PMC3499251

[32] He HH, Meyer CA, Shin H, Bailey ST, Wei G, Wang Q, et al. Nucleosome dynamics define transcriptional enhancers. Nature genetics. 2010;42(4):343–347. doi: 10.1038/ng.545 20208536 PMC2932437

[33] Wiechens N, Singh V, Gkikopoulos T, Schofield P, Rocha S, Owen-Hughes T. The chromatin remodelling enzymes SNF2H and SNF2L position nucleosomes adjacent to CTCF and other transcription factors. PLoS genetics. 2016;12(3):e1005940. doi: 10.1371/journal.pgen.1005940 27019336 PMC4809547

[34] Zuin J, Dixon JR, van der Reijden MI, Ye Z, Kolovos P, Brouwer RW, et al. Cohesin and CTCF differentially affect chromatin architecture and gene expression in human cells. Proceedings of the National Academy of Sciences. 2014;111(3):996–1001. doi: 10.1073/pnas.1317788111 24335803 PMC3903193

[35] Girton JR, Johansen KM. Chromatin structure and the regulation of gene expression: the lessons of PEV in Drosophila. Advances in genetics. 2008;61:1–43. doi: 10.1016/S0065-2660(07)00001-6 18282501

[36] Swagatika S, Tomar R. Modulation of Epigenetics by Environmental Toxic Molecules. Advances in Molecular Toxicology. 2016;10:361–389. doi: 10.1016/B978-0-12-804700-2.00008-8

[37] Ding X, Lin X, Zhang B. Stability and folding pathways of tetra-nucleosome from six-dimensional free energy surface. Nature communications. 2021;12(1):1–9. doi: 10.1038/s41467-021-21377-z 33597548 PMC7889939

[38] Song F, Chen P, Sun D, Wang M, Dong L, Liang D, et al. Cryo-EM study of the chromatin fiber reveals a double helix twisted by tetranucleosomal units. Science. 2014;344(6182):376–380. doi: 10.1126/science.1251413 24763583

[39] Liu T, Wang Z. Reconstructing high-resolution chromosome three-dimensional structures by hi-C complex networks. BMC bioinformatics. 2018;19(17):39–50. doi: 10.1186/s12859-018-2464-z 30591009 PMC6309071

[40] Wang Y, Zhang Y, Zhang R, van Schaik T, Zhang L, Sasaki T, et al. SPIN reveals genome-wide landscape of nuclear compartmentalization. Genome biology. 2021;22(1):1–23. doi: 10.1186/s13059-020-02253-3 33446254 PMC7809771

[41] Gong Y, Lazaris C, Sakellaropoulos T, Lozano A, Kambadur P, Ntziachristos P, et al. Stratification of TAD boundaries reveals preferential insulation of super-enhancers by strong boundaries. Nature communications. 2018;9(1):1–12. doi: 10.1038/s41467-018-03017-1 29416042 PMC5803259

[42] Nuebler J, Fudenberg G, Imakaev M, Abdennur N, Mirny LA. Chromatin organization by an interplay of loop extrusion and compartmental segregation. Proceedings of the National Academy of Sciences. 2018;115(29):E6697–E6706. doi: 10.1073/pnas.1717730115 29967174 PMC6055145

[43] Krietenstein N, Abraham S, Venev SV, Abdennur N, Gibcus J, Hsieh THS, et al. Ultrastructural details of mammalian chromosome architecture. Molecular cell. 2020;78(3):554–565. doi: 10.1016/j.molcel.2020.03.003 32213324 PMC7222625

[44] Oveisi M, Shukla M, Seymen N, Ohno M, Taniguchi Y, Nahata S, et al. iNucs: inter-nucleosome interactions. Bioinformatics (Oxford, England). 2021; p. btab698. doi: 10.1093/bioinformatics/btab698 34623394 PMC8652021

[45] Krietenstein N, Rando OJ. Mesoscale organization of the chromatin fiber. Current opinion in genetics & development. 2020;61:32–36. doi: 10.1016/j.gde.2020.02.022 32305817

[46] Kulis B, Jordan MI. Revisiting k-means: New algorithms via Bayesian nonparametrics. arXiv preprint arXiv:11110352. 2011.

[47] Langmead B, Salzberg SL. Fast gapped-read alignment with Bowtie 2. Nature methods. 2012;9(4):357–359. doi: 10.1038/nmeth.1923 22388286 PMC3322381

[48] Li H, Handsaker B, Wysoker A, Fennell T, Ruan J, Homer N, et al. The sequence alignment/map format and SAMtools. Bioinformatics. 2009;25(16):2078–2079. doi: 10.1093/bioinformatics/btp352 19505943 PMC2723002

