# Supplementary figures and images for: NucleoMap: A computational tool for identifying nucleosomes in ultra-high resolution contact maps
Source: PLoS Comput Biol. 2022 Jul 14;18(7):e1010265. doi: 10.1371/journal.pcbi.1010265 (PMC9321407; doi:10.1371/journal.pcbi.1010265)

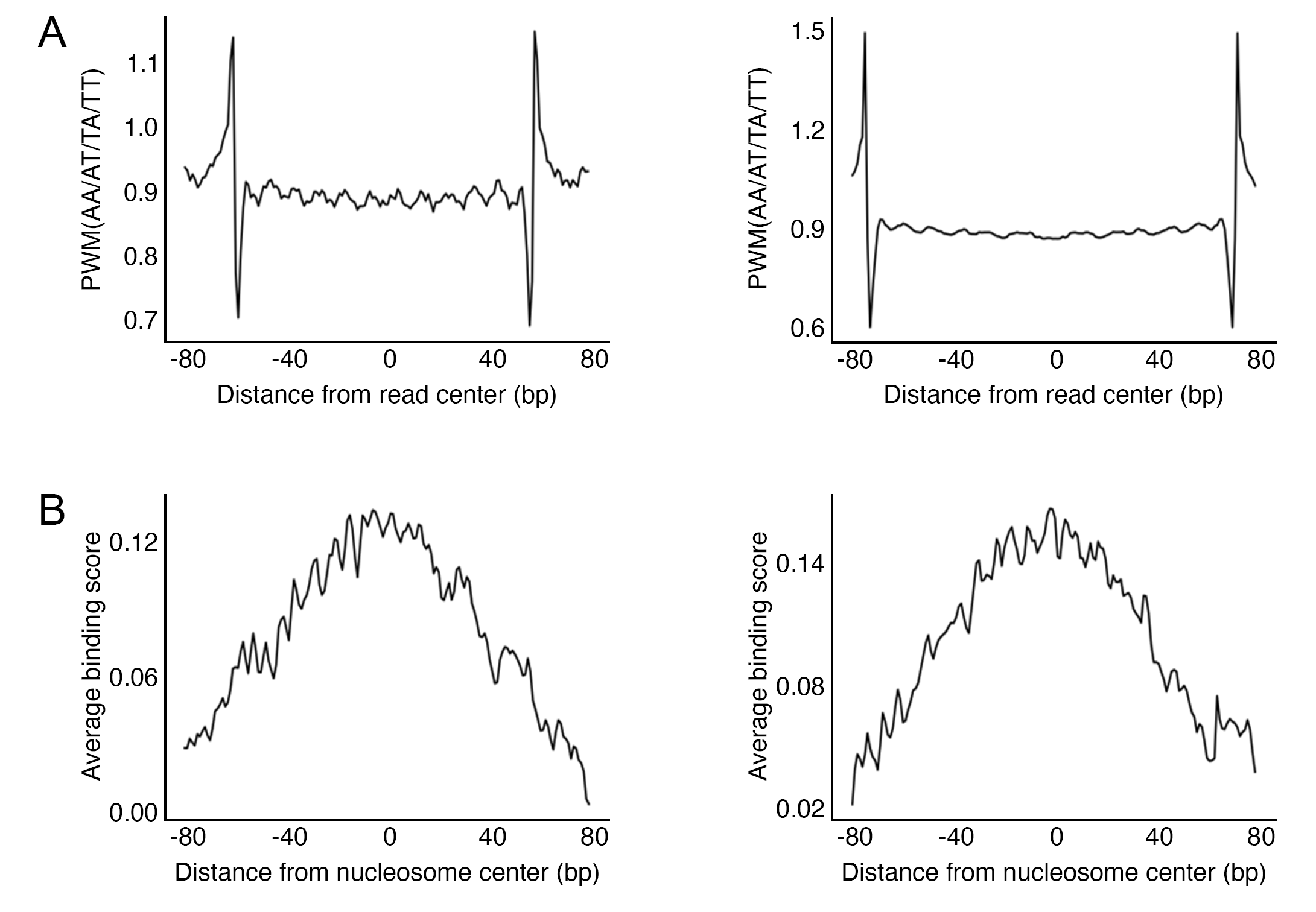

Supplement: S1 Fig — A. Dinucleotide PWMs of yeast (left) and hESC (right). The dinucleotide PWMs in yeast and hESC reflect similar nucleosome binding preference of ∼10bp periodic AA/AT/TA/TT 2-mers in the two cell lines. B. Average nucleosome-binding scores around experimentally identified nucleosomes (left) and computational identified nucleosomes (right) in yeast. Peaks of motif-based nucleosome-binding scores centered at both experimentally and computationally identified nucleosomes indicate that the nucleosome-binding score defined in NucleoMap effectively captures the nucleosome sequence preference. (TIF) [file pcbi.1010265.s001.tif]

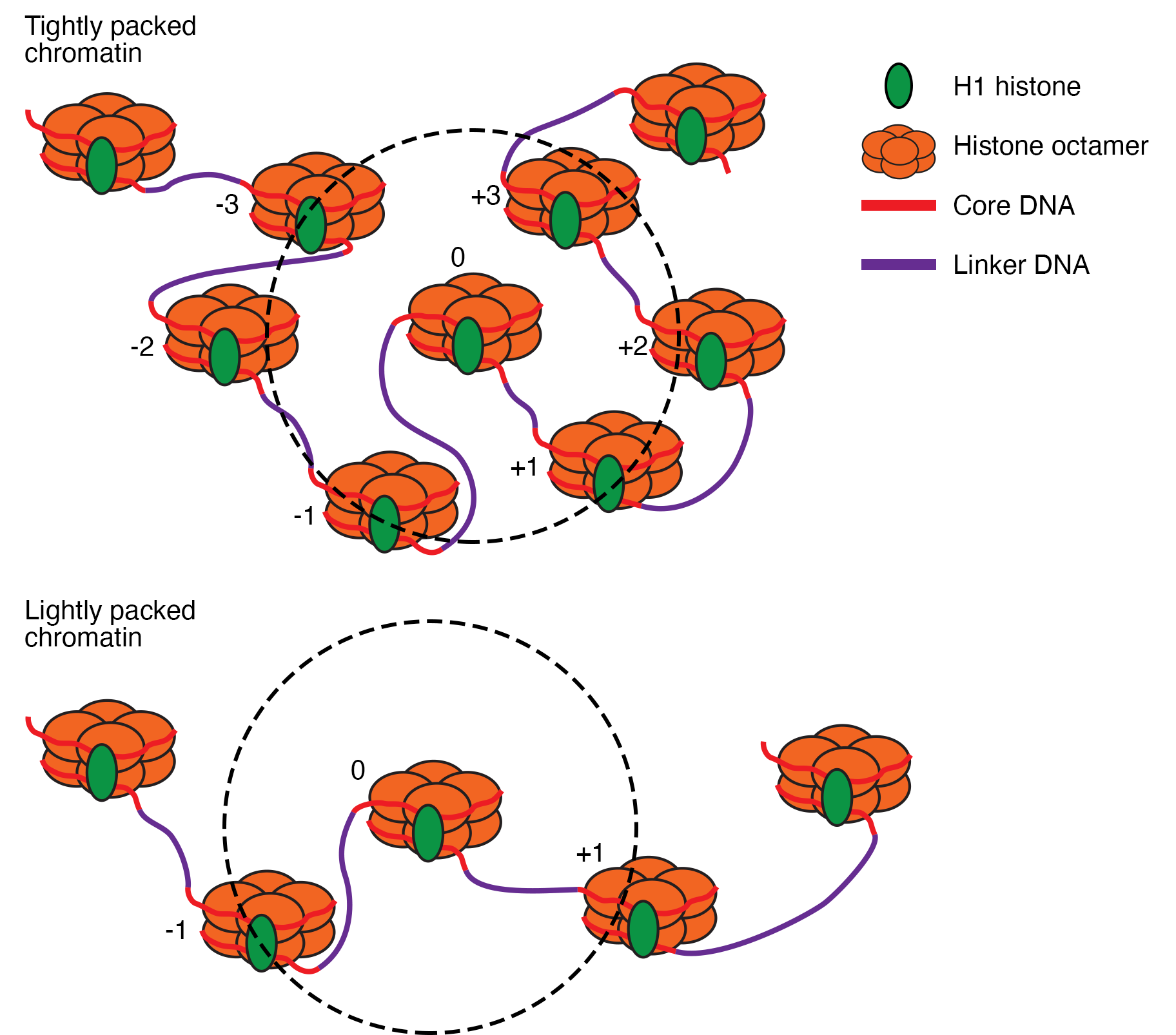

Supplement: S2 Fig — Frequencies of inter-nucleosome contacts correlate with the spatial distance between nucleosome pairs. In tightly packed chromatin, the neighborhoods of central nucleosomes involve more adjacent nucleosomes (±3 nucleosomes in the example) and thus forming longer average contact distances, whereas in lightly packed chromatin, fewer nucleosomes are involved in the neighborhoods of the central nucleosomes, forming shorter average contact distances. (TIF) [file pcbi.1010265.s002.tif]

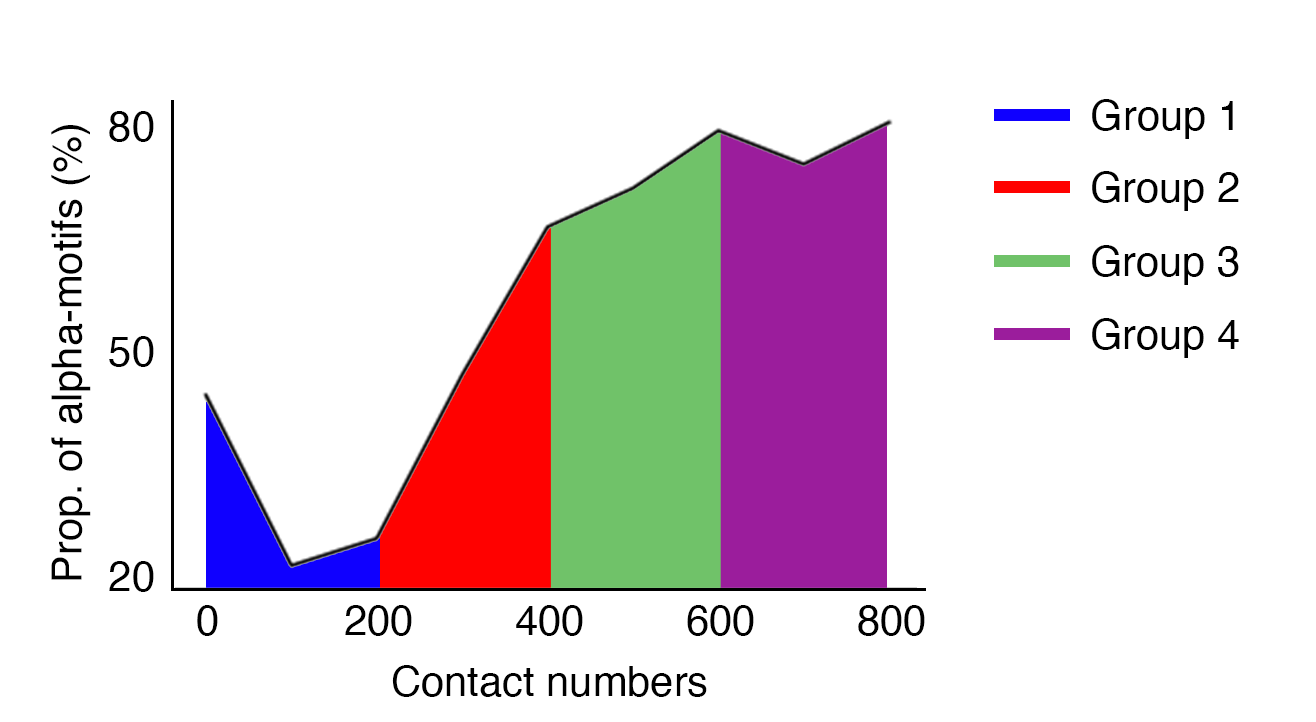

Supplement: S3 Fig — Neighborhood with more contacts tends to have higher percentages of α-tetrahedrons. The nucleosomes are divided into four groups according to their local contact numbers. Within each group, the slope (the trend of forming α-tetrahedrons with respect to contact numbers) is approximately constant. (TIF) [file pcbi.1010265.s003.tif]

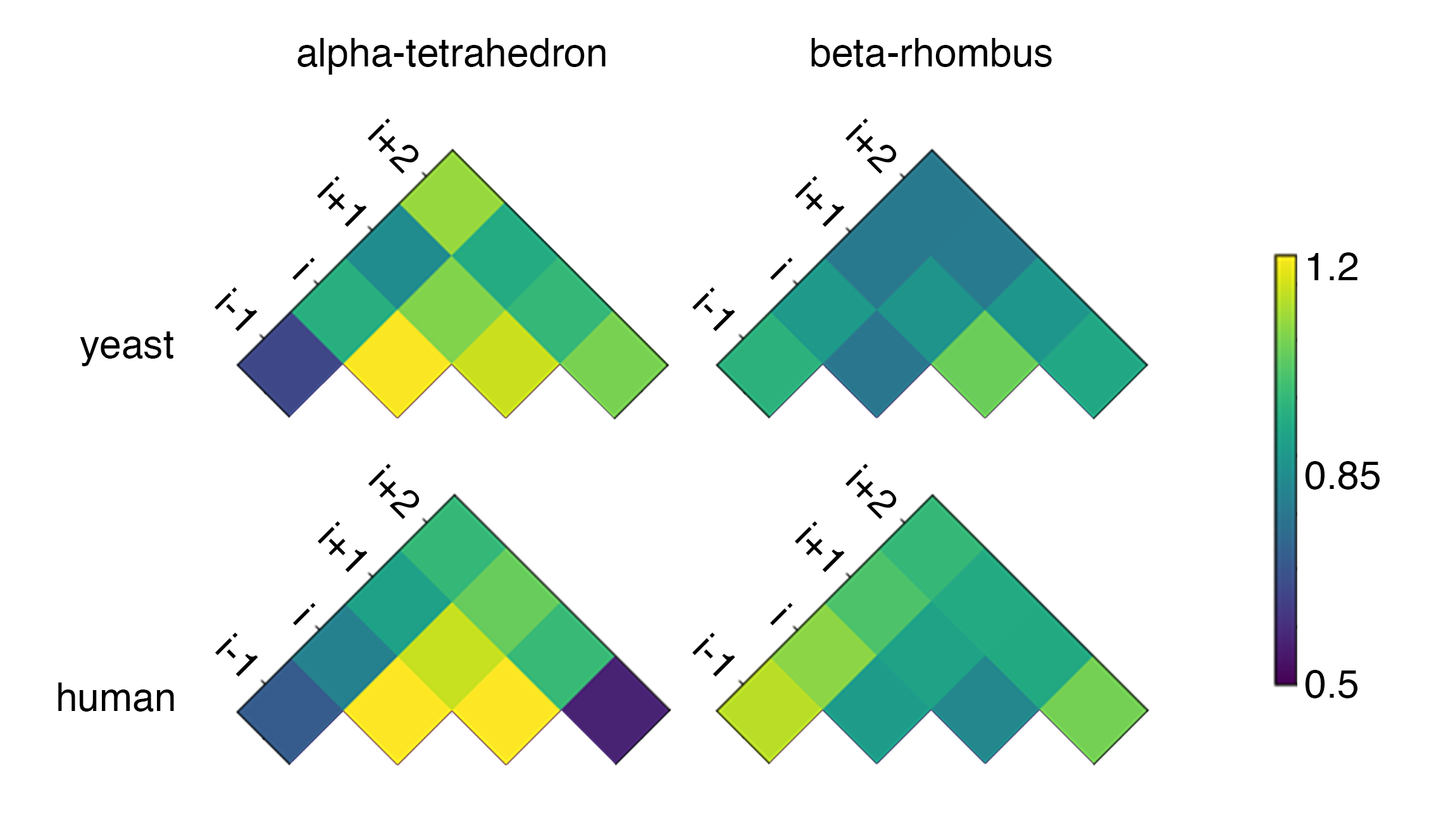

Supplement: S4 Fig — Nucleosomes of α-tetrahedrons and β-rhombuses predicted by machine learning models in human embryonic stem cells have consistent local contact patterns with yeast. Here average local contact maps of the two tetra-nucleosome motifs between the i−1-th and the i+2-th nucleosomes are presented. Values in the contact maps are OE normalized. (TIF) [file pcbi.1010265.s004.tif]

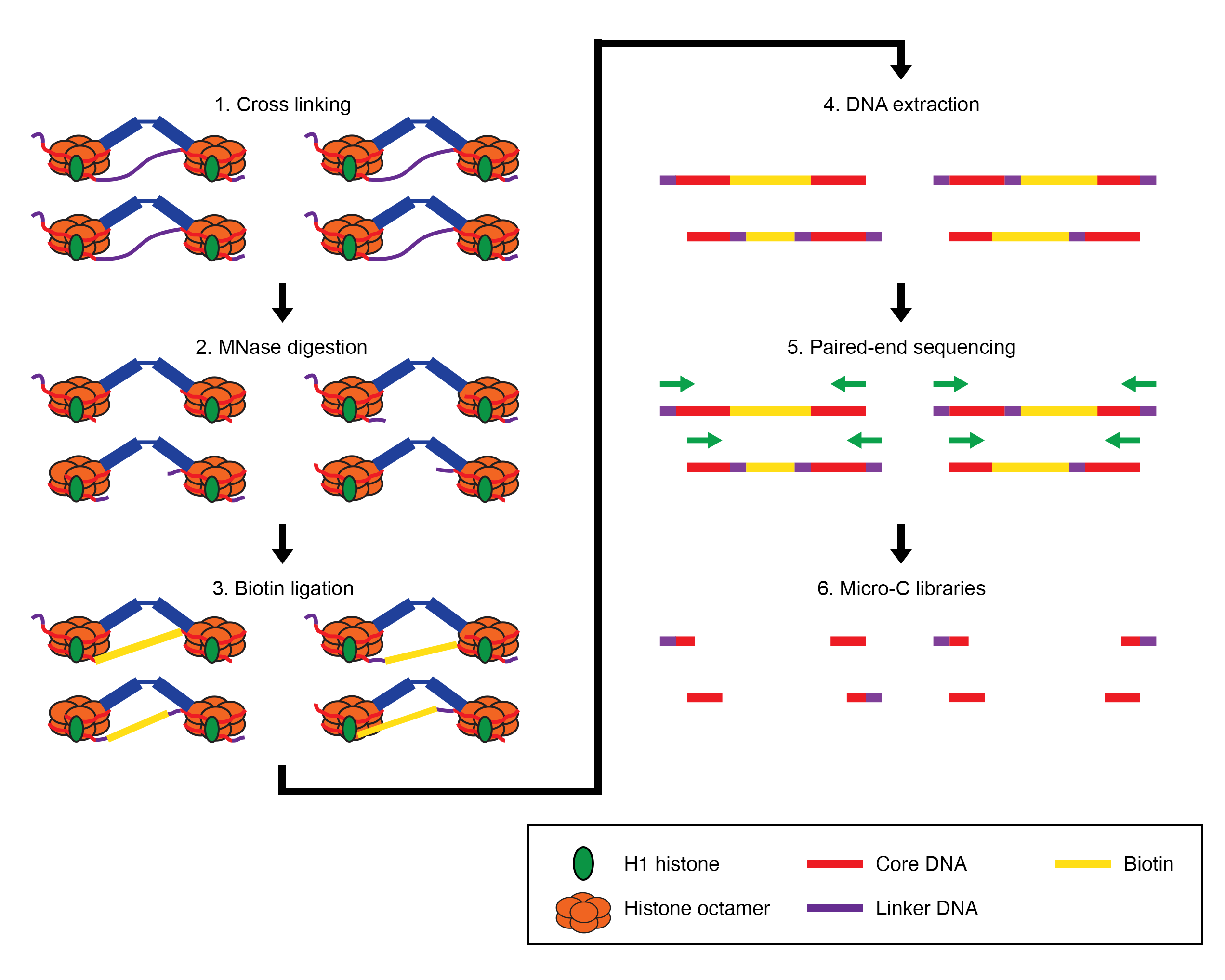

Supplement: S5 Fig — The Micro-C libraries are generated as follows. 1. Fix chromatin with formaldehyde. 2. Digest linker DNA in crosslinked chromatin with MNase. In this step, MNase does not strictly digest linker DNA. A small fraction of linker DNA is remained, while core DNA in some nucleosomes is partially digested. 3. Ligate the ends of remaining DNA with biotin according to their spatial proximity. 4. Digest protein and extract ligated DNA contacts. 5. Pair-end sequencing of the contacts. 6. Micro-C libraries are generated, containing the ∼50bp sequence of one end of every nucleosome. (TIF) [file pcbi.1010265.s005.tif]

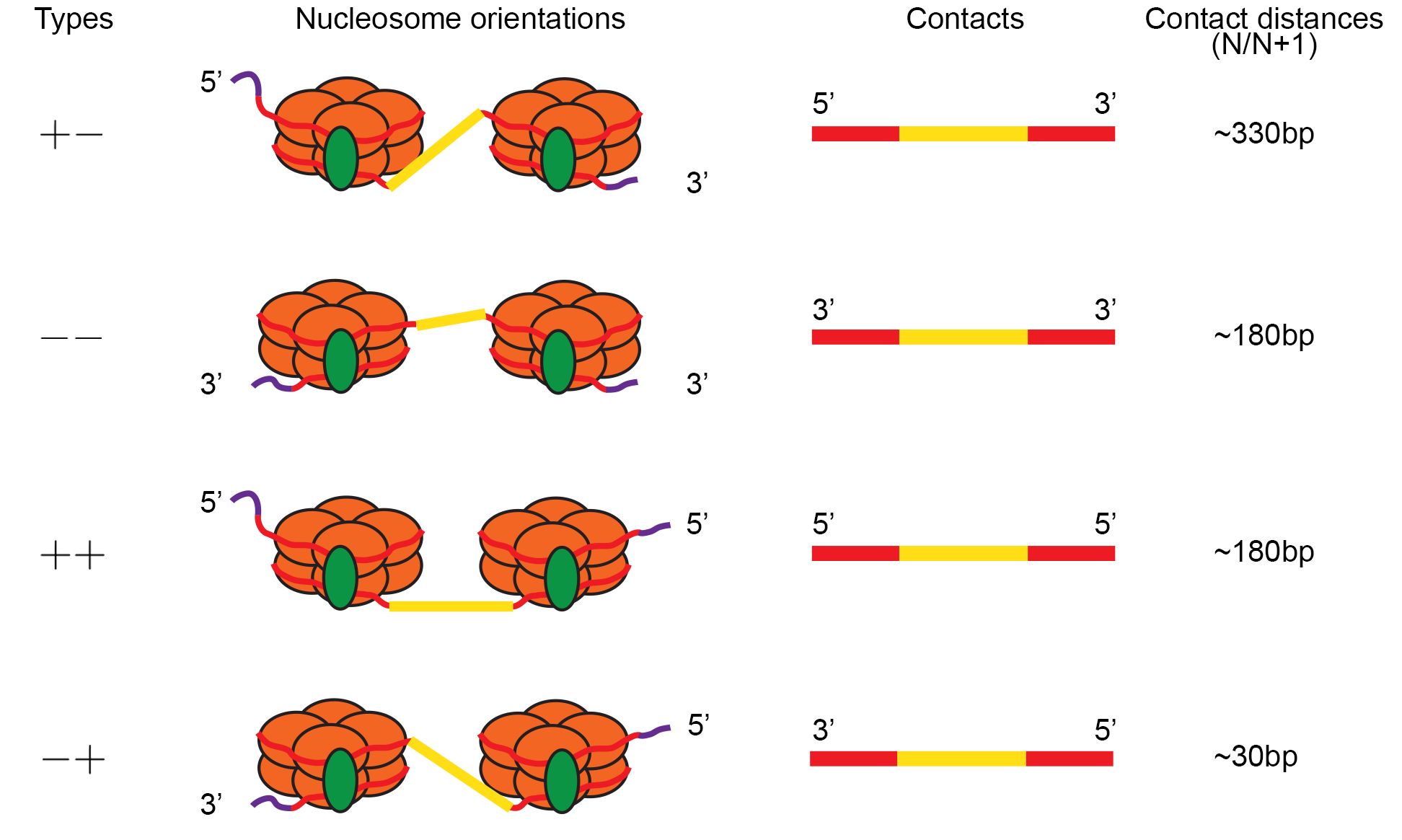

Supplement: S6 Fig — Biotin ligation is formed between the closest ends of core DNAs wrapping around the nucleosome pairs. Depending on the nucleosome orientation, four types of contacts can be formed between two nucleosomes, namely, +−, −−, ++, and −+. Even if they anchor the same nucleosome pairs (e.g., contacts between N/N+1 nucleosomes), different contact types vary in contact distance measured by the genomic distance between two ends of a contact. (TIF) [file pcbi.1010265.s006.tif]

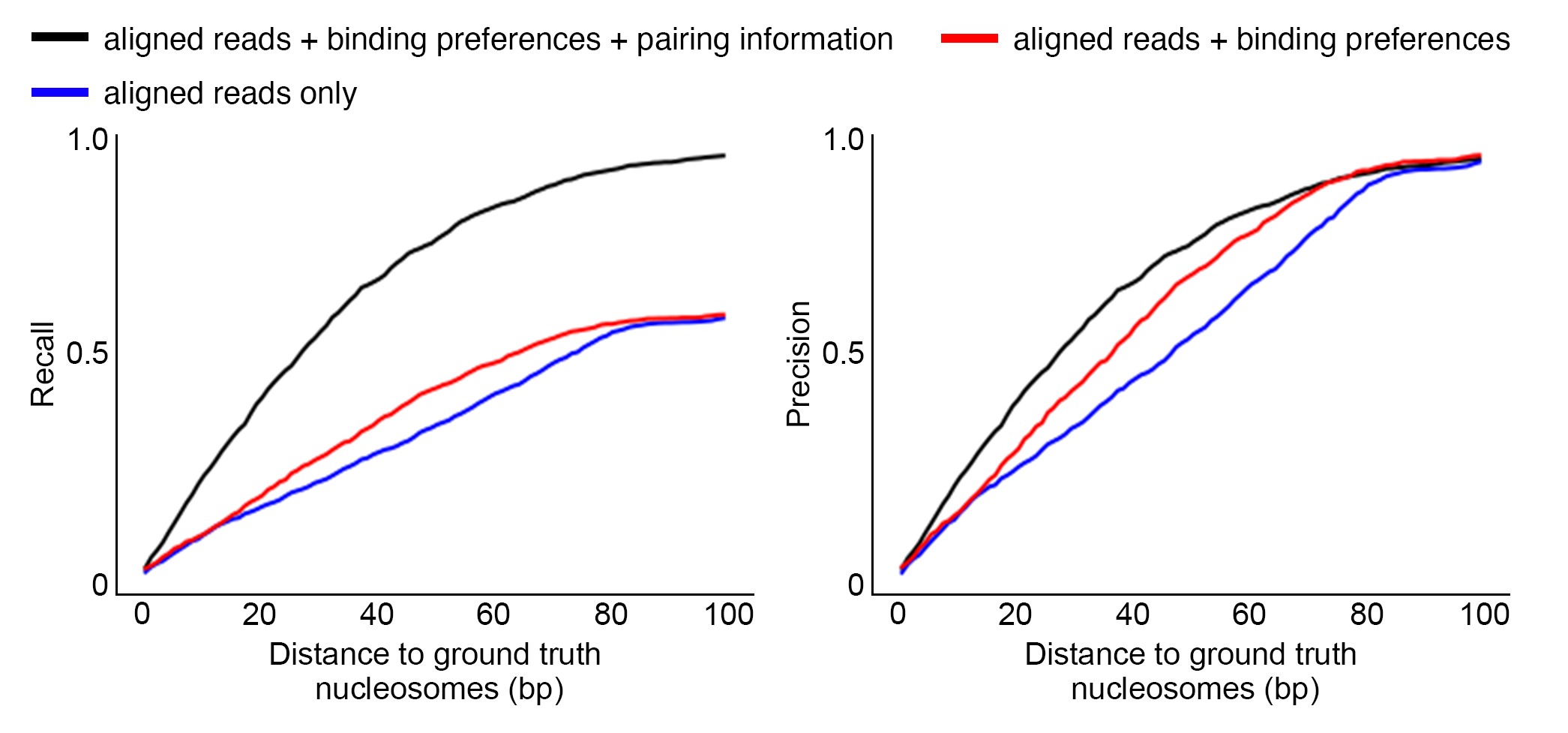

Supplement: S7 Fig — The aligned reads play the most crucial role in detecting nucleosomes, accounting for the largest areas under the curves for both precision and recall. The pairing information significantly improves the recall of NucleoMap, and it also contributes to the precision of our method. The binding preferences improve the precision and recall when dt is small, suggesting that it helps locat nucleosomes more accurately. (TIF) [file pcbi.1010265.s007.tif]
